# Differential effects of Th1, monocyte/macrophage and Th2 cytokine mixtures on early gene expression for glial and neural-related molecules in central nervous system mixed glial cell cultures: neurotrophins, growth factors and structural proteins

**DOI:** 10.1186/1742-2094-4-30

**Published:** 2007-12-18

**Authors:** Robert P Lisak, Joyce A Benjamins, Beverly Bealmear, Liljana Nedelkoska, Bin Yao, Susan Land, Diane Studzinski

**Affiliations:** 1 Department of Neurology, 8D University Health Center, Wayne State University School of Medicine, 4201 St Antoine, Detroit, MI, 48210, USA; 2 Department of Immunology and Microbiology, Wayne State University School of Medicine, 540 E Canfield Avenue, Detroit, MI 48201, USA; 3 Department of Biochemistry and Molecular Biology, Wayne State University School of Medicine, 540 E Canfield Avenue, Detroit, MI 48201, USA; 4 Applied Genomics Technology Center, 5107 Biological Sciences, Wayne State University, 5047 Gullen Mall, Detroit MI 48202, USA; 5 Center for Molecular Medicine and Genetics, Wayne State University School of Medicine, 540 E Canfield, Detroit, MI 48201, USA; 6 Department of Surgery, 6C University Health Center, Wayne State University School of Medicine, 4201 St. Antoine, Detroit MI, US 48201 USA; 7 Department of Surgery, William Beaumont Hospital, 3601 W. Thirteen Mile Rd., Royal Oak MI 48073 USA

## Abstract

**Background:**

In multiple sclerosis, inflammatory cells are found in both active and chronic lesions, and it is increasingly clear that cytokines are involved directly and indirectly in both formation and inhibition of lesions. We propose that cytokine mixtures typical of Th1 or Th2 lymphocytes, or monocyte/macrophages each induce unique molecular changes in glial cells.

**Methods:**

To examine changes in gene expression that might occur in glial cells exposed to the secreted products of immune cells, we have used gene array analysis to assess the early effects of different cytokine mixtures on mixed CNS glia in culture. We compared the effects of cytokines typical of Th1 and Th2 lymphocytes and monocyte/macrophages (M/M) on CNS glia after 6 hours of treatment.

**Results:**

In this paper we focus on changes with potential relevance for neuroprotection and axon/glial interactions. Each mixture of cytokines induced a unique pattern of changes in genes for neurotrophins, growth and maturation factors and related receptors; most notably an alternatively spliced form of trkC was markedly downregulated by Th1 and M/M cytokines, while Th2 cytokines upregulated BDNF. Genes for molecules of potential importance in axon/glial interactions, including cell adhesion molecules, connexins, and some molecules traditionally associated with neurons showed significant changes, while no genes for myelin-associated genes were regulated at this early time point. Unexpectedly, changes occurred in several genes for proteins initially associated with retina, cancer or bone development, and not previously reported in glial cells.

**Conclusion:**

Each of the three cytokine mixtures induced specific changes in gene expression that could be altered by pharmacologic strategies to promote protection of the central nervous system.

## Background

The pathogenesis of lesions in the central nervous system (CNS) of patients with multiple sclerosis (MS) represents the end product of several immune processes. Secretion of cytokines by infiltrating inflammatory cells as well as by endogenous glia contributes directly and indirectly to the pathogenesis of the MS lesions [1,2]. Whether the initial lesions in CNS white matter affect oligodendrocytes [3] or activated microglia in normal appearing white matter (NAWM) [4] is still not clear, but inflammatory cells are part of active and chronic active lesions, the type I and II lesions of Luchinnetti and Lassmann, as well as the type III and type IV lesions, which are considered primarily degenerative or toxic lesions of oligodendrocytes [5]. It is also increasingly clear that cytokines are directly and indirectly involved in inhibition of lesion formation.

There has been renewed interest in the pathologic changes in axons in the white matter [6,7] as well as lesions in the gray matter in patients with MS [8,9]. These changes, which can be seen in the earliest lesions, have lead to the view of MS as being, at least in part, a degenerative disease of the CNS. Activated endogenous glia, particularly microglia, and perhaps infiltrating inflammatory cells may also contribute to the pathogenesis of lesions in other more classically degenerative diseases of the CNS such as Alzheimer's disease, Parkinson's disease and amyotrophic lateral sclerosis (ALS). [10-13]. Since MS has a neurodegenerative component and because much of the permanent disability in MS results from axonal and neuronal dysfunction, irreversible damage and cell loss [14,15], there is increasing interest in treatments that are able to attenuate neuronal damage and perhaps allow for regeneration, so called "neuroprotection" [16-18]. Several studies have emphasized the potential for the immune system to provide neuroprotection and encourage repair in experimental immunopathogenic disorders of the CNS [19-21] and peripheral nervous system (PNS) [22] as well as in traumatic [23,24] and degenerative diseases [25,26]. While much of the work on neuroprotection is in animal models, there is some indirect evidence for neuroprotection provided by immunomodulatory therapy in patients with MS [27-31]. In addition to protection of axons and neurons and stimulation of regeneration of damaged axons, it is naive to consider neurons and axons in isolation from glial cells. Thus there is also a need to identify factors that could inhibit demyelination and/or protect oligodendrocytes and enhance oligodendrocyte precursor maturation. Additionally the effects of cytokines, chemokines and growth factors on astrocytes and microglia, which undoubtedly play several roles in neuroprotection, axonal outgrowth and synaptogenesis as well as development of neurons, need consideration and study.

The development of MS lesions, as well as inhibition of lesion formation and reparative processes, involves complex interactions among mixtures of CNS cells. In addition single infiltrating inflammatory cells and glial cells do not secrete single cytokines, and glial cells do not respond by producing single growth factors or modifying only one of their many cell functions. MS is a highly complex disease with regards to basic immune and inflammatory mechanisms involving the systemic immune system as well the CNS. As such, complex techniques need to be applied in MS research [32,33] so as to avoid oversimplification. For these reasons we have carried out a series of experiments to examine the effects of different cytokine mixtures, typical of Th1 and Th2 lymphocytes, as well as monocyte/macrophages (M/M) on early gene expression in mixed glial cell cultures. This approach also eliminates the confounding effects of infiltrating inflammatory cells. We chose to employ microarray technology, which allows one to simultaneously examine the regulatory effect of these mixtures of cytokines on a vast number of genes. Thus one may not only see effects on a large number of genes but may find regulatory effects on unanticipated genes. To identify genes more likely to be directly affected by the cytokine mixtures, we chose to start by examining the effect on gene expression at 6 hours *in vitro *(early gene expression).

Our rationale is to use genomics to identify the significant early changes in gene expression in the interactive mixed glial cell environment, with subsequent experiments focused on identifying the specific cell types responsible for those changes. Because of the large number of genes regulated by these cytokine mixtures and issues of manuscript length we reported the effects on genes of immune system related molecules in an earlier separate report [34] and divided other data into this paper with neurotrophins, other growth factors and structural proteins, and a third paper looking at ion channels, neurotransmitters and their receptors, mitochondria, signaling, transcription factors, and molecules involved in apoptosis, among others. In the first report we noted that expression of at least 814 genes of 7,985 analyzed were changed by a minimum of 2 fold by one or more of the cytokine mixtures at 6 hours (early gene response) *in vitro *when compared to control cultures. In this report and another paper in progress, we present results of regulation of genes for molecules more classically related to glial and axonal/neuronal cells. We are aware that the assignment of molecules to one or the other such broad categories is arbitrary since many of these molecules act in the immune system as well as in the CNS. In addition, some molecules could be classified under any one of several categories in this report. As examples, BIG-2 could be considered a neuronal protein but is also an adhesion molecule; deleted in colon cancer (DCC) is a cancer-related protein but also a neuronal protein.

## Methods

The methodology has been described in detail in the prior paper [34].

### Mixed CNS glial cell cultures

As described in the first report, mixed CNS glial cell cultures were obtained from neonatal rat brain using a modification the so-called "shake-off" technique[35,36]. The time for removal of microglia by adherence to plastic was reduced to 1 hour prior to plating on poly-lysine coated flasks following "shake-off". Cells were maintained in defined medium containing 2% fetal bovine serum for 6–8 days, then treated with the cytokines. Culture composition was determined by employing indirect immunofluorescence (IF) antibodies to phenotypic markers for different cells types: glial acidic fibrillary protein (GFAP) for astrocytes [37] (Chemicon, Temecula, CA); galactolipids (GalL) for oligodendrocytes [37,38], A2B5 for oligodendrocyte precursors [39] (ATCC, Bethesda, MD); ED-1 for microglia [40] (Serotec, Raleigh, NC); Th1.1 for fibroblasts [41] and some astrocytes [42] (in glial cultures), anti-neurofilament heavy (NFh) for neurons [43] and anti-factor VIII for endothelial cells (Dako Corporation, Carpinteria, CA). To identify cells expressing major histocompatibility complex class II molecules (MHC class II) we employed mouse anti-MHC class II (Serotec).

### Cytokine mixtures

Th1: Recombinant rat interleukin-2 (IL-2) and recombinant rat interferon-γ (IFN-γ) (R&D Systems, Inc, Minneapolis), recombinant rat tumor necrosis factor-α (TNF-α; BD PharMingen, San Diego, CA) and recombinant rat granulocyte-colony stimulating factor (G-CSF; PeproTech, Rocky Hill, NJ).

M/M: Recombinant rat IL-1α and IL-1β, recombinant rat IL-6, recombinant rat IL-12p40 (all from R&D Systems, Inc) and recombinant rat TNF-α. These cytokines are considered to be proinflammatory products of what have recently been termed M1 macrophages or microglia [44].

Th2: Recombinant rat IL-4, recombinant rat IL-5, recombinant rat IL-10 (all from R&D Systems, Inc), recombinant rat G-CSF and purified porcine transforming growth factor-β1 (TGF-β1; R&D Systems, Inc). It has been suggested that in the cognate immune system, TGF-β1 is the product of a population of T-cells called regulatory T-cells (Treg cells) and TGF-β is important in differentiation of these Treg cells. Treg cells are phenotypically characterized as CD4+/CD25high+/Fox3 [45,46] and also secrete IL-10 [47].

Cytokine mixtures contained 10 ng/ml of each of the constituent cytokines as is typically employed in many *in vitro *studies of cytokine biology and as employed by our group [34]. Cultures were incubated with mixtures of Th1, Th2, or M/M cytokines or additional medium (control) for 6 hours. Three separate experiments each consisting of control, Th1, M/M and Th2 stimulated cultures (a total of 12 cultures) were performed.

### Cytotoxicity

Since certain cytokines have been reported to induce death of oligodendrocytes and their precursors [48-50], we examined the cytokine-induced effect on cell death in mixed CNS glial cell cultures by incubating cultures from 6 hours to 4 days with the cytokine mixtures. Cell death was determined by uptake of 0.4% trypan blue [51].

### RNA extraction

After 6 hours of incubation with cytokine mixtures or additional medium, cultures were washed and RNA was extracted employing TRIzol (Gibco BRL, Grand Island, NY) followed by Qiagen RNeasy kits (Qiagen, Valencia, CA). The RNA was quantitated at A_260 nm _and the quality was assessed by at A_260 nm_/A_280 nm_. The 28S/18S ratio was assessed using a Bioanalyzer 2100 (Agilent Technologies, Wilmington, DE) and was >1.7 for all samples.

### Gene array analysis

Biotin-labeled RNA fragments were produced from 5 μg of total RNA by first synthesizing double-stranded cDNA followed by a transcription reaction and a fragmentation reaction. A hybridization cocktail which contained fragmented cRNA, probe array controls (Affymetrix), bovine serum albumin and herring sperm DNA was hybridized to the Affymetrix rat RG-U34A microarray at 45°C for 16 hours. The probe array was then washed and bound biotin-labeled cRNA detected with streptavidin phycoerythin conjugate. Subsequent signal amplification was performed employing biotinylated anti-streptavidin antibody. The RG-U34A chip contains 7,985 genes. Control and 3 cytokine-incubated cultures from one experiment were analyzed with one gene chip for each sample and three separate experiments using different cultures were analyzed. The transcriptional profile data have been submitted to the GEO repository, with accession number GSE9659.

### Data analysis

Gene array data were analyzed by several methods to determine which genes were upregulated and downregulated compared to control cultures for each series of cultures, as well as comparing the average of the replicates from each of the 3 separate sets of experiments. Affymetrix data were analyzed with dChip v1.2 to calculate gene expression values [52]. We analyzed values from 3 separate experiments employing the ANOVA GeneSpring, Agilent Technologies, Santa Clara, CA) comparing Th1, M/M and Th2 with control. While multiple testing analyses that compare all 7,985 genes at different levels of stringency using the Bonferoni and false discovery rate (FDR) at p < 0.05 are statistically most rigorous, at these high levels of stringency, there were very few changes that reached statistical significance. In order to increase sensitivity and allow identification of potentially important biologic changes, we employed a lower level of stringency. [34] While this approach is likely to result in more false positive results, it allows identification of gene changes in gene expression and potentially protein expression that can later be verified by quantitative real time polymerase chain reaction (QRT-PCR) and Western blotting respectively. In these screening studies at a single time point, we have arbitrarily chosen to represent as probably significant those genes in which the mean expression was >2 fold (upregulation) or <-2 fold (downregulation) compared to expression in controls (p < 0.2) [34]. We believe this is reasonable given that our experiments consisted of biological replicates that are prone to greater variability than experimental replicates. The recent literature suggests that a 2-fold cut-off using the Affymetrix platform produces a low false positive rate [53].

### Quantitative real time-polymerase chain reaction (QRT-PCR) expression analysis

Expression of message for BDNF and NT3 was analyzed by QRT-PCR on an ABI 7500 Fast System, using ABI Taqman rat specific gene expression assays. RNA was extracted as above and reverse transcribed. Relative expression levels were calculated with GAPDH as the internal reference, using the delta-delta Ct method [54]. The values from the treated cultures were compared to those from control. Those ratios were averaged for the three experiments, then expressed as fold changes in the treated cultures relative to control for comparison with the gene array results. Each PCR value represents the average from 2–5 separate experiments.

## Results

### Mixed CNS glial cell cultures

As described in our earlier report, cultures consisted of approximately 35% each oligodendrocytes and astrocytes and 10% microglia. The remaining cells were glial cell precursors including A2B5 oligodendrocyte precursors. There were no endothelial cells or neurons. Viability was >98% in both control and cytokine stimulated cultures at all time points (6 hours to 5 days), demonstrating the lack of cytotoxicity under these experimental conditions.

### Overview of cytokine effects on early gene expression

Our previous paper [34] described changes in genes for proteins predominantly associated with the immune system including major histocompatibility molecules, some adhesion and extracellular matrix molecules, cytokines and chemokines and their receptors, complement components and others. Because of our interests in the direct effects of cytokines on endogenous CNS cells affecting the production of factors important in oligodendrocyte health and differentiation/myelination and axonal and neuronal function, in this paper we compare the effects of the different cytokine mixtures on gene expression of genes associated with glial cells and neurons/axons. The results are shown in Table 1 with changes in various categories summarized briefly below, but presented in more detail in the Discussion.

**Table 1:** Summary of changes in gene expression in neurotrophins, growth factors, related receptors and structural proteins

**NEUROTROPHINS AND RECEPTORS**		**Th1**	**M/M**	**Th2**
M55293	trkB	-3.05*		-2.7*
E03082	NT3	-4.15***	-2.71***	-4.73****
AI030286	brain derived neurotrophic factor	-4.19*		
AA944973	NGFR p75 precursor	-4.26**		
S62933	trkC, alternatively spliced	-12.13***	-7.01***	
AF023087	NGF-inducible IA		2.30***	2.25*
X67108	brain derived neurotrophic factor			2.11*
M86742	pre-pro NT4 protein			-2.62**
Y07559	NT 4/5			-2.63**
**GROWTH/MATURATION FACTORS AND RECEPTORS**				
AI070577	insulin-like growth factor receptor 5	3.66**		
Z14120	platelet-derived growth factor alpha (PDGF)	2.99****	3.20***	2.23***
M32167	vascular endothelial growth factor (VEGF)	2.39*		3.13**
D79215	fibroblast growth factor 10	2.17*	2.59***	2.44*
M81183	insulin-like growth factor 1 (IGF-1)	-2.02**		
E0178	TGF-alpha	-2.09**		
M90660	insulin receptor-related receptor alpha	-2.88**		
X12748	epidermal growth factor precursor	-3.48****		-2.53***
U37101	granulocyte colony stimulating factor 3 (GCSF)		3.24****	
D10106	prepropeptide PDGF A chain		2.73***	2.31***
D64085	fibroblast growth factor 5		2.18*	
M18416	early growth response 1		2.03*	
X07285	basic fibroblast growth factor		-2.06*	
U66470	cell growth regulatory with EF-hand domain		-2.07*	
AA818970	endothelin receptor type B			2.70****
AB008908	fibroblast growth factor 14			2.64*
AF014827	c-fos induced growth factor (VEGF D)			2.24**
S54008	fibroblast growth factor receptor 1			2.01***
U02320	neuregulin 1			-2.17**
AA875664	GCSF signaling molecule, mitochondria-associated			-2.24****
M17960	insulin-like growth factor II (somatomedin A)			-2.95**
AI137657	platelet-derived growth factor receptor alpha			-3.04*
**HORMONES, RECEPTORS AND RELATED MOLECULES**				
U94321	growth hormone secretagogue receptor	3.09*	3.13***	
M54987	corticotropin releasing hormone	2.17*		2.40*
U55836	parathyroid hormone receptor		-2.39**	
U25803	luteinizing hormone, beta subunit	-2.57***	-2.14**	-2.18*
M34083	prolactin receptor	-2.86***	-2.69***	
M63296	luteinizing hormone receptor	-2.92*		
AA799498	natriuretic peptide precursor	-3.09**		
M27408	follicle stimuating hormone beta	-3.21*		-3.30*
Z33403	luteinizing hormone receptor	-3.77**	-2.80*	
Z83757	growth hormone receptor	-4.06*	-3.81*	-4.58*
Al235978	aldosterone receptor	-6.13		
S49003	short isoform growth hormone receptor		2.78*	
U25802	luteinizing hormone, beta subunit		-2.79***	
L40030	placental growth factor		-3.33**	
X01454	thyroid stimulating hormone, beta subunit		-3.55***	
U41183	growth hormone releasing hormone		-5.09*	
M19304	prolactin receptor		-5.81**	
AA893618	glucocorticoid receptor			-2.64*
U92469	gonadotropin-releasing hormone receptor			-6.06**
AA946542	prolactin-like protein D			-6.09*
Al180410	prolactin-like protein C			-6.70***
**CELL ADHESION AND EXTRACELLULAR MATRIX MOLECULES**				
S61868	syndecan 4	2.8*	3.5**	
AA818894	proteoglycan core peptide	2.2*		
U56859	perlecan	-3.29*		
S61868	syndecan 4		2.9*	
U16845	neurotrimin		2.76*	2.68*
U09401	tenascin c			3.92**
U15550	tenascin c			2.3*
Al071104	glypican 3			2.04**
MI5797	nidogen (entactin)			-2.02*
D50568	proteoglycan, bone marrow			-2.12*
AJ011811	claudin 7			-2.12*
U35371	BIG-2			-2.47**
X63143	syndecan 3 (neuroglycan)		-2.44**	-2.35**
AA8000059	syndecan 4			-2.9**
Al639167	biglycan (weakly similar to bone/cartilage p.g.)			-3.57**
**CONNEXINS**				
M76532	connexin 37 (CXN-37)	2.76**		
M59936	connexin-31 (CXN-31)	2.03**		
AF022136	connexin 40 (CXN-40)		-2.82*	
**NEURONAL RELATED PROTEINS**				
M74223	VGF nerve growth factor inducible	3.04***	3.79***	2.25***
AF031430	syntaxin 7	2.09***		
AF056704	synapsin 3	-2.18*		-4.27***
S96418	activin receptor IIA	-3.41*		
U69702	activin receptor-like kinase 7	-3.62**		
AA818677	neurofilament, heavy polypeptide	-4.16*		
Y16563	bassoon	-4.88*		
X17682	MAP 2		3.33**	
U22952	neuroligin		2.49***	
X66840	MAP 1A		-2.07*	
X06655	synaptophysin		-2.13***	
X06655	synaptophysin 2		-2.19****	
M64488	synaptotagmin		-2.20****	
U71924	synaptotagmin XII		-4.56*	
AF041246	hypocretin (orexin)		-5.88*	-3.87***
L20820	syntaxin 3			-2.43***
X95286	semaphorin 3a			-2.94***
**RETINAL PROTEINS**				
X52376	rds/peripherin		2.09***	2.15***
Z46957	rhodopsin		-4.64***	-5.14**
**CANCER RELATED PROTEINS**				
AF036760	breast cancer 1	-2.28*	-2.52*	-2.05*
U68725	deleted in colorectal cancer (rat homolog)	-4.05**		
U32081	deleted in malignant brain tumors 1		-6.02***	
M32475	carcinoembryonic antigen			-4.58*
**BONE-RELATED PROTEINS**				
Y07704	Best 5 protein	39.49****	7.02**	
Y07704	Best 5 protein	31.46***	7.41*	
AA892798	USAG (uterine-sensitized associated gene 1)			-2.10*

Values represent averages of fold changes from three separate experiments for each cytokine mixture compared to control. **** p < 0.01; *** p < 0.05; ** p < 0.10; * p < 0.20

Some of the gene classifications are arbitrary as many proteins could be listed and considered under two or more different categories. As noted, this is a series of screening experiments and therefore the table and figures were prepared using the criteria of > 2 fold (increased expression) or <-2 fold (decreased expression) with a p value of <0.2 for one or two replicates of the gene transcript [34]. Values for unidentified genes (EST) showing changes by these criteria are not presented.

**Figure 1:**
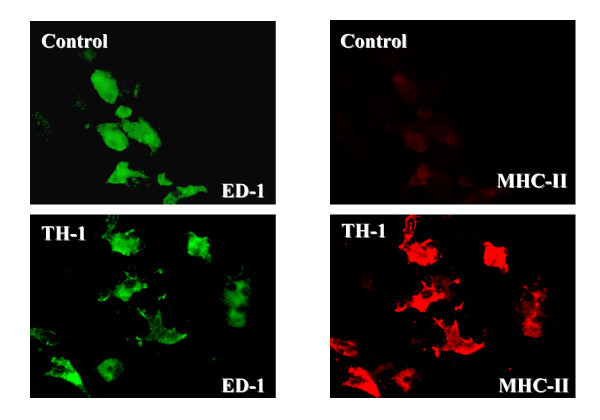
**Effect of Th1 cytokines on microglia**. CNS mixed glial cultures were incubated with Th1 cytokines or additional culture medium for 4 days. Cultures were fixed with 4% paraformaldehyde for 10 minutes, washed and then incubated with mouse anti-ED-1 (IgM) and mouse anti-rat MHC class II (IgG) followed by Alexa 488-conjugated goat anti-mouse IgM and Cy 3-conjugated donkey anti-mouse IgG. Cultures were examined for indirect immunofluoresence employing a Leitz Orthoplan 2 fluorescent microscope. The Th1 treated microglia (ED-1+ cells) have a different appearance when compared to those incubated with additional medium (control). Control microglia do not express MHC class II whereas Th1 treated microglia strongly express MHC class II molecules.

**Figure 2:**
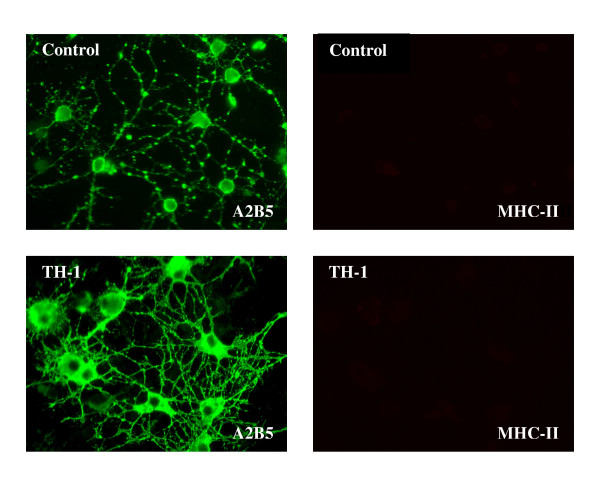
**Effect of Th1 cytokines on oligodendrocyte precursors**. CNS mixed CNS glial cultures were incubated with Th1 cytokines or additional medium and fixed as in Figure 1. Cultures were then incubated with mouse A2B5 (IgM) and mouse anti rat MHC class II (IgG) followed by Alexa 488-conjugated goat anti-mouse IgM and Cy 3-conjugated donkey anti-mouse IgG. Cultures were examined by indirect immunofluoresence as above. Compared to control, the Th1 treated A2B5+ oligodendrocyte precursors have a much more mature appearance including more extensive process formation although the cells still express A2B5, but do not express MHC class II.

In view of the pleiotropic effects of the cytokine mixtures on gene expression, we would predict changes in cellular phenotype and function. Although these changes will be examined in more detail in subsequent studies, two examples illustrating effects of the Th1 cytokine mixture on microglia and oligodendrocyte precursors are shown in Figures 1 and 2. By 4 days *in vitro *microglial cells have changed their appearance when compared to unstimulated cells and now expresses MHC class II on their surface. Oligodendrocyte precursors still express A2B5 on their surface but have begun to show much more elaborate and extensive processes.

### Neurotrophins and receptors

Th2 cytokines induced upregulation of the gene for brain derived neurotrophic factor (BDNF), but in general the cytokine mixtures had downregulatory effects on the genes for neurotrophins and their receptors. The most marked downregulation was found for an alternatively spliced form of trkC with both Th1 and M/M cytokines. (Table 1; -12 fold and -7 fold, respectively). For validation of gene array results, expression of message for BDNF and NT3 was analyzed by QRT-PCR. Results from the two methods were in general agreement, with the exception of Th2 effects on NT3 at 6 hours; subsequent QRT-PCR analysis at 24 hours showed a 3.44 fold upregulation, supporting the conclusion that Th2 increased the expression of NT3 at 6 hours (Table 2).

**Table 2:** Effects of cytokines on BDNF and NT3 gene expression: comparison of gene array and QRT-PCR results

**ACCESSION ID**	**GENE**	**TREATMENT**	**FOLD CHANGE**	
			**6 hr array**	**6 hr PCR**

Al030286, X67108	BDNF	Th1 cytokines	-4.19	-1.14
		MM cytokines	no change	1.09
		Th2 cytokine	2.11	2.08
				
E03082	NT3	Th1 cytokines	-4.15	-2.5
		MM cytokines	-2.71	-1.09
		Th2 cytokines	-4.73	2.13

CNS glial cultures were treated with the cytokine mixtures and analyzed by Affymetrix gene array or QRT-PCR, as described in Methods. The gene array values are the averages of 3 experiments; the QRT-PCR values are the averages of 3–5 experiments.

### Growth and maturation factors and receptors

There are overlaps in classification of several proteins as cytokines or growth factors, often considered as one or the other depending on the effect being studied. Th1, M/M and Th2 cytokine mixtures had complex and variable effects on genes for many proteins in this category of molecules (Table 1). However there was no effect on glial derived neurotrophic factor (GDNF) or GDNF family members (neurturin, artemin or persephin) or their receptors, although sequences for many of these genes are present on the microarray chip we employed in these experiments.

### Hormones and hormone receptors

The different cytokine mixtures also had effects on some genes for hormonal trophic and release factors and receptors for these and other related hormones. Th1 and particularly M/M cytokines had both up and downregulatory effects on several genes within this category whereas Th2 cytokines had more restricted effects (Table 1).

### Cell adhesion and extracellular matrix molecules

In our initial paper, we noted changes in this class of molecules known to be involved in immune related interactions. In the present paper, we present data on molecules that may be involved in neuronal/glial interactions. Th1, M/M cytokines as well as Th2 cytokines had regulatory effects on a small number of genes for cell adhesion and extracellular matrix (ECM) molecules including several proteoglycans (Table 1).

### Connexins

Th1 cytokines upregulated the genes for connexin 31 and connexin 37, while M/M cytokines downregulated the gene for connexin 40.

### Myelin associated proteins

Although sequences of the genes specific for several myelin associated proteins are present on the microarray chip, we found no effects on genes for myelin-associated proteins at 6 hours *in vitro *when compared to control cultures.

### Neuronal related proteins

Although there are no neurons in our cultures, we observed cytokine-induced regulation of genes for neuronal/axonal related proteins (Table 1). The genes for several other proteins associated with neurons were regulated by the different cytokine mixtures and are listed under adhesion and extra-cellular matrix molecules. In a subsequent paper, we describe changes in genes for many ion channels, peptides and transmitters and receptors associated with neurons but which are also known to be expressed by glial cells.

### Retinal proteins

M/M cytokines downregulated the gene for rhodopsin, and Th2 cytokines upregulated the gene for rat retinal degradation slow/peripherin 2 (rds/peripherin 2) (Table 1).

### Cancer related proteins

We have noted an effect of the cytokines on expression for genes for two proteins first described in cancer biology, rat homologs for breast cancer 1 (BCR1), called BCRA1, and deleted in colorectal cancer (DCC), both of which have subsequently been reported to be present in neurons. In addition there was marked downregulation by M/M cytokines of the gene for carcinogenic embryonic antigen (CEA), and marked downregulation by the Th2 cytokines of a gene which is deleted in some gliomas. (Table 1)

### Bone-related proteins

Th1 and M/M cytokines markedly upregulated the gene for BEST 5, a protein involved in bone modeling, and Th2 cytokines downregulated uterine-sensitized associated gene 1 (USAG1), a gene for an inhibitor of the bone morphogenic protein family.

## Discussion

Since the immune system seems capable of providing protection and promoting regeneration in the CNS and PNS, it is reasonable to examine the roles of cytokines in these reparative and protective processes. Cytokines themselves may act directly as growth and development factors in the nervous system [55,56], whether secreted by invading inflammatory cells or produced by endogenous glial cells. In addition inflammatory/immune system cells secrete classic neurotrophins and other factors that promote neuronal and oligodendrocyte growth, developmental and maintenance [57-59]. It has been known for many years that some cytokines are able to induce secretion of neuronal growth factors by endogenous cells of the CNS and PNS [26,60,61]. Cytokine induction of growth factors, including neurotrophins, by glial cells could serve as a major source of such factors contributing to repair and remyelination. Cytokines also likely induce oligodendrocyte and oligodendrocyte precursor cell damage and/or inhibit recovery by down regulating growth factors and their receptors. One would expect a differential effect of different types of cytokines, in particular Th1 versus Th2 cytokines and perhaps cytokines associated with monocytes/macrophages and microglia. This has been shown for microglia [44,62]. In the following discussion we will highlight some of the genes regulated and discuss the implications of changes in the proteins specified by these genes for MS as well as other neurologic diseases.

Recently, potential roles for both T regulatory (Treg) and Th17 cells have been identified in EAE [63-68], and cytokines secreted by these cells may also influence the pathogenesis of lesions in multiple sclerosis [69,70] and optical spinal multiple sclerosis [71]. The profile of these cells under some conditions is partially known; for example, Th17 cells secrete IFN-γ and the chemokine CCL20 [72]. More investigations will be needed to identify whether the effects of cytokines secreted by the proinflammatory Th17 cells more closely resemble those of Th1 cells than of Th2 cells or macrophages [73,74].

### Neurotrophins and receptors

With the exception of a 2-fold upregulation of the gene for brain derived neurotrophic factor (BDNF) by Th2 cytokines, most of the genes for the neurotrophins and their receptors were either downregulated by the different cytokine mixtures or not affected in comparison to control cultures. In preliminary studies employing quantitative real time polymerase chain reaction (QRT-PCR), we have detected Th2 cytokine induced upregulation of the gene for BDNF at both 6 and 24 hours. Although Th2 cytokines upregulated the gene for BDNF, they induced a -2.6-fold downregulation of genes for pre-pro NT4/5, a response potentially inhibitory for neuroprotection, depending upon the timing of the downregulation and the cell involved [75]. Downregulation of the genes for BDNF, neurotrophin 3 (NT 3), trkB (receptor for BDNF as well as for NT3), pre-pro NT 4/5, and an alternatively spliced gene for trkC (receptor for NT3) by Th1 cytokines, and of NT3 and trkC by M/M cytokines, if accompanied by decreased levels of their proteins, would be detrimental to neurons/axons and oligodendrocytes as well as inhibitory to regeneration [76-80].

The gene for NGF-inducible protein 1A was interestingly upregulated 2-fold by both M/M and Th2 cytokine mixtures. While inflammatory cells, including Th2 lymphocytes, produce neurotrophins [57,81,59], cytokines produced by the same infiltrating cells, particularly Th1 and M/M cells, could inhibit the production of several of these neurotrophins by both the endogenous glial cells and the infiltrating cells. Perhaps of equal importance, these cytokine mixtures seem to downregulate the genes for receptors for several of these neurotrophins. BDNF and trkB have been detected in MS lesions [82], while an increase in gene expression for NT3 and NGF2 has been reported in marginal zones of two active lesions in MS [83]. The presence and concentration of the different factors as well as the sequence of their appearance may be important in neuroprotection and remyelination. It is quite clear that more than one factor is important for neuroprotection and regeneration as well as for oligodendrocyte health and OPC maturation into oligodendrocytes that are able to myelinate axons [84,85]. Both the combinations of neurotrophins as well as concentrations and timing of appearance and suppression of the neurotrophins and their receptors are likely to be critical. In addition it is known that the effects of a particular neurotrophin on a cell is in part determined by the balance of expression of its protein tyrosine kinase receptors, such as trkB, and the low avidity p75 NGFR. [86,87] Th1 cytokines downregulated the gene for NGFR p75 precursor protein, which not only binds all members of the NT family and triggers cell death pathways, but also seems to be involved in the common pathway signaling for Nogo, myelin associated glycoprotein (MAG) and oligodendrocyte myelin glycoprotein (OmGp), in the inhibition of neurite outgrowth by myelin [88].

### Other growth and maturation factors and receptors

We observed changes in expression of a large number of genes for neuropoietic cytokines [34] and for non-neurotrophin growth factors, important in different phases of oligodendrocyte as well as neuronal/axonal development. Some of these deserve comment as they may relate to pathogenesis and recovery in MS and in other neurological diseases.

Th1 cytokines induced downregulation of the gene for TGF-α which signals via the epithelial growth factor receptor (EGFR) [89], as well as down regulating the gene for EGF precursor. Interestingly Th2 cytokines also downregulated expression of the gene for EGF precursor at 6 hours *in vitro *(Table 1). TGF-α has been reported to be present in astrocytes [90] and has effects on astrocyte function [91,92] and development and function of neurons [89,93], as well as acting as a growth factor for tumors of astrocyte [94] and non-CNS origin [95]. It seems to have an antiapoptotic effect [92] and in combination with IFN-γ upregulates cyclooxygenase2 (Cox 2) [96]. Thus Th1 cytokines, which include IFN-γ, by downregulating production of TGF-α as well as EGF in inflammatory diseases such as MS, would inhibit protection/development of certain neuronal populations. However, this same activity in tumors would tend to inhibit tumor growth and survival.

EGF is critical in the development of the CNS, being one of the more important growth factors for neuronal development, acting on neural progenitor cells, as well as in myelination [97-101].

Perhaps of equal importance is the down regulation of bFGF (also called FGF2) by M/M cytokines, since this growth factor appears important to some but perhaps not all stages of neuronal and oligodendrocyte development and protection [102,100-107]. FGF2 acts as a mitogen for OPCs. Thus if M/M cytokines decreased expression of FGF2, this would tend to inhibit OPC proliferation. As noted later, some syndecans appear to bind to FGF2. Upregulation of the gene for FGF2 has been reported in some MS lesions [69] and FGFR-1 has been reported present at the margins of chronic active and chronic inactive lesions in MS [83]. There was also regulation of the genes for other FGFs and receptors. EGF in combination with bFGF is important in early stages of neuronal and stem cell proliferation and later development [108,109].

All three cytokine mixtures increased gene expression for platelet-derived growth factor alpha (PDGF α) by 2 to 3-fold, and M/M and Th2 cytokines similarly upregulated the gene for prepropeptide PDGF A chain. Th2 cytokines downregulated the gene for PDGF-Rα. At early stages of OPC development, upregulation of PDGF-α and its receptor would favor repair by serving as a mitogenic stimulus, but persistent upregulation could inhibit ultimate maturation and myelination, and downregulation of PGDF-Rα at the correct time would favor transition from a precursor stage to a more mature stage [110-112]. In addition PDGF acts in concert with the chemokine Gro-α (CXCL1) to induce OPC proliferation although persistence of these two factors seems to prevent OPCs from undergoing further maturation [113,114]. In our earlier report we described Th1 cytokine upregulation of the gene for Gro-α [34].

We observed effects of the cytokine mixtures on genes for several other growth factors important for oligodendrocyte maturation and myelination as well as for neuronal survival and development including neuregulin-1, insulin-like growth factor (IGF-1) and one of its receptors/binding proteins, IGF-R5 [115].

As with neurotrophins, downregulation of neuropoietic cytokines and other growth factors could inhibit certain phases of oligodendrocyte development and myelination, as well as favor neurodegeneration. However, at other stages inhibition of these same growth factors might be necessary for OPC and neuronal maturation.

Neuregulins are a group of factors which are important in CNS development, with multiple actions including precursor proliferation, cell migration, synaptogenesis and control of certain aspects of myelination. Made in neurons and perhaps in glia, neuregulins have the capacity to bind to other cells in the nervous system and thus influence a large number of functions of several CNS cell types [116-121]. We observed downregulation of the gene for neuregulin type I induced by the Th2 cytokine mixture. Lack of type II neuregulins (glial growth factor 2, GGF2) has been reported in MS lesions by one group of investigators [122] whereas another group found increased levels when compared to non-MS white matter [123].

Insulin like growth factors (IGFs) are important in neuronal and oligodendrocyte development [124-127]. There are conflicting studies on the effects of IGF-1 on both the clinical course and remyelination in animals with EAE [128-131]. As summarized in the Table 1, Th1 cytokines down regulated the gene for IGF-1 but upregulated the gene for IGFR-5, one of several receptors for IGF-1. Th2 cytokines downregulated the gene for IGF-2 (somatomedin), which can be found in CNS and PNS and may have a protective role in inhibiting TNF-α-induced oligodendrocycte death [132,133].

Vascular endothelial growth factor (VEGF) is an important factor in vascular biology particularly in angiogenesis [134,135], and may have neurotrophic effects [136-138]. VEGF may contribute to inflammation within the CNS and has been detected in acute and chronic active MS plaques [139,140]. Th1 and Th2 cytokines both induced a 2 to 3-fold upregulation of the gene for VEGF.

### Hormone and hormone receptors

Th1 and M/M cytokines induced upregulation of the growth hormone secretagogue receptor, and both Th1 and Th2 cytokines upregulated corticotrophin releasing hormone, but in general Th1 and M/M cytokines had downregulatory effects on genes in this category. If the cytokines had a similar effect on certain neuronal populations, these findings might explain the overall downregulatory effect of acute and chronic inflammation on normal endocrine function and the increase in the corticosteroid response in these same conditions. Prolactin regulates oligodendrocyte precursor proliferation [141] and if downregulation of the gene for prolactin receptor that we observed in response to Th1 and M/M cytokines results in reduced availability of receptor, there would be reduced ability of such cells to develop and supply cells to replace injured and dead oligodendrocytes as a source of remyelination. Th2 cytokines induced downregulation of two different prolactin-like proteins but not prolactin or its receptor.

### Adhesion and extracellular matrix proteins

The cytokine mixtures had varying effects on the genes for adhesion proteins and proteins associated with ECM, including many arbitrarily classified as immune-molecule related and reported in our earlier paper. Important among those were several integrins and selectins, CD44 and several matrix metalloproteins [34]. In this study, we observed changes in regulation of genes for several other adhesion-related proteins that may be important in oligodendrocytes, oligodendrocyte precursor cells (OPCs), neurons, axons as well as in axonal-glial interactions.

The proteoglycans are a group of glycoproteins with covalently bound sulfated glycosaminoglycan (GAG) chains which have been found in virtually all tissues and are widely expressed on most cells types represented in the CNS and PNS [142,143]. They are important in cell-cell interactions as well as interactions of cells with the ECM. The syndecans are proteoglycans and are type I transmembrane proteins with either heparan sulfate (HS) or chondroitin sulfate (CS) chains [142]. In our studies the expression of syndecan-4 was upregulated by Th1 and M/M cytokine mixtures, and downregulated by Th2 cytokines. Syndecan-4 has been found on various glial cells and has a mix of HS and CS bound side chains[142,144]. The ligands for syndecan 4 have not been fully defined but other syndecans and possibly syndecan-4 are known to bind to several growth factor including FGF2 [142,144] and TGF-β, as well as to ECM molecules such as heparin-binding growth-associated molecule (HB-GAM or pleiotrophin) and midkine [142]. In this study we demonstrated down regulation of the gene for FGF2 at 6 hours in response to incubation with M/M cytokines, upregulation of fibroblast growth factor receptor-1 (FGFR-1) by Th2 cytokines (see below and Table 1) and in our earlier report regulation of genes for several binding and signaling molecules associated with TGF-β by the different cytokine mixtures [34]. Syndecan -1, the gene for which was not affected in our study, binds to tenascin-C, an adhesion molecule. The gene for tenascin-C was upregulated by Th2 cytokine mixtures. It has been shown that TGF-β(a component of the Th2 cytokine mixture) and FGF2 upregulate tenascin-C [145]. Tenascin-C is one of a series of extracellular molecules which bind to several proteoglycans including, as noted, syndecan-1, as well as syndecan 3 (neuroglycan), a chondroitin sulfate proteoglycan. Syndecan-1 is found in several organ systems; in the CNS syndecan-1 is likely a neuronal membrane component while neuroglycan is CNS specific, and expressed on neurons and astrocytes [142]. Tenascin-C, which can bind to ng-CAM/Li and NCAM on neurons [146], inhibits adhesion to collagen [147], and is found in developing and injured CNS [148,149]. It inhibits oligodendrocyte migration [150,151] and inhibits axonal outgrowth [152]. Tenascin-C knockout mouse embryos fail to develop normal cell migration [153] emphasizing that normal development requires a balance between positive and negative signals. Finally tenascin-C, which is found in normal myelinated tissue, is reduced in acute MS lesions and found in reactive astrocytes in chronic lesions [154].

Glypicans, proteoglycans that are membrane bound, essentially have only HS side chains. Glypican-3 is found in the nervous system although its exact localization is not clear. There is some evidence that it helps regulate activity of insulin-like growth factor-II (IGF-II), perhaps by binding to IGF-II. Other cell surface molecules and ECM also serve as ligands for glypican-3 [142]. A loss of function mutation of glypican-3 is associated with Simpson-Golabi-Behmel (SGB) syndrome, which has pre and postnatal overgrowth and risk of embryonic tumors such as Wilms tumors and neuroblastomas, the latter a tumor of neuronal origin [155]. Interestingly over expression of IGF-II has been associated with Beckwith-Wiedmann syndrome, a disorder similar to SGB [156,157]. We observed upregulation of the gene for glypican 3 by mixtures of Th2 cytokines. As noted above, Th2 cytokines downregulated the gene for IGF-II.

There is strong evidence, predominately in vitro, that proteoglycans in their interactions with growth factors, ECM and cell surface molecules are important in the regulation of cell migration and proliferation in the CNS, as well as neurite outgrowth and guidance and synaptogenesis. Proteoglycans may be involved in inhibition of Schwann cell entry into the CNS and inhibit neurite extension at the damaged CNS/PNS interface [158], and inhibition of proteoglycans allows improved recovery in a spinal cord injury model [159]. Given the potential importance of proteoglycans in the CNS, it is of interest that cytokine mixtures differentially up- and downregulated the expression of genes for several of these proteoglycans and some of their ligands.

We also observed cytokine regulation of several adhesion molecules that are members of the immunoglobulin superfamily. Neurotrimin is a neuronal adhesion molecule that seems to inhibit axonal outgrowth, which is important in development of CNS and sympathetic nervous system [160,161]. M/M and Th2 cytokine mixtures upregulated the gene for this protein, which at certain stages of CNS disease such as MS might result in inhibition of neuronal regeneration, but at other stages could be important in repair by helping direct appropriate connections.

The gene for contactin 4 (also called BIG-2), a member of the TAG-1 subgroup of axon-associated cell adhesion molecules (AxCAMs; members of the Ig superfamily), was downregulated by Th2 cytokines [162]. Contactins, which are expressed by neurons/axons and oligodendrocytes, complex with a protein called contactin associated protein (Caspr) and are found at paranodal regions of axon and glial interaction [163]. Contactins are uniformly distributed along bare axons, and TAG1 is found at paranodes with Caspr and is downregulated along myelinated axons [164]. While there are no studies of the distribution of contactins in MS lesions, there is a study of the distribution of Caspr on axons. Axons in the center of demyelinated lesions do not express Caspr. Paranodal regions of remyelinated axons express Caspr, and axons in border zones of lesions express Caspr, but not in the normal paranodal pattern [165]. Contactin 4 is not known to directly interact with Caspr and the exact nature of its ligand is not clear. However it seems to be important in axonal/neurite development [162,166].

We also detected Th2 cytokine downregulation of claudin 7, a member of the claudin family, tetraspan proteins involved in tight junction formation. Claudin 7 is expressed on CD4 T-lymphocytes, and downregulated in tumor progression, but not previously identified in glia [167,168]. Other claudins are expressed by endothelial cells and choroid plexus epithelial cells [169,170], as well as myelin forming cells and in myelin itself [171,172].

### Connexins

Connexins are proteins that are part of gap junctions between cells, which allow for direct communication between these cells [173,174]. Some connexins are found in glial cells and some seem to be important in myelination [175]. The genes for connexins 31 and 37 were upregulated by Th1 cytokines, and connexin 40 was downregulated by M/M cytokines. Connexins 31 and 40 seem to be involved in pathways subserving hearing [176-178].

### Myelin associated proteins

At 6 hours, we did not detect any effect of the cytokine mixtures on the genes for any myelin associated proteins such as myelin basic protein (MBP), proteolipid protein (PLP), myelin associated glycoprotein (MAG), oligodendrocyte myelin glycoprotein (OmGp) or claudin 11 (oligodendrocyte specific protein; Osp) although, as noted, the genes for these proteins and other myelin associated proteins are on the gene chips. This may relate to the use of a 6 hour incubation, and follow-up studies examining additional time points are clearly needed to further investigate the effect of cytokines on the genes for these proteins. It is known that IL-2 effects oligodendrocyte maturation and expression of MBP [55,179].

### Neuronal related proteins

Although there are no neurons in our cultures, we detected regulation of expression of genes for several important proteins associated with neurons. Some have already been discussed under other categories, including adhesion molecules. Others that are known to be expressed in glial cells as well as neurons, such as some of the ion channels, transmitters and receptors, will be presented in a subsequent paper. In the following section, we comment on genes for other proteins that may be of considerable importance in the pathogenesis of several diseases of the CNS, as well as in neuroprotection and repair mechanisms.

Reduction in cerebrospinal fluid (CSF) levels of hypocretin (orexin) are associated with classical narcolepsy [180] as well as other disorders of sleep [181], and reduced hypocretin positive neurons have also been described in narcolepsy [182,183]. Hypocretin also seems to be involved in energy metabolism and drinking behavior [184,185]. Hypocretin containing neurons in the hypothalamus have widespread projections in the CNS including to the spinal cord [186,187] M/M cytokines downregulated the gene for hypocretin receptor 2 (-5.9-fold), one of two receptors for hypocretin, in glial cell cultures, as did Th2 cytokines (Table 1). Levels of hypocretin itself have been reported as normal in the CSF in most if not all studies of MS, but nothing is known about expression of receptors for hypocretin in the CNS of patients with MS [181,183]. If downregulation of hypocretin receptors in neurons could be shown *in vitro *in response to cytokines and *in vivo*, particularly in neurons of the hypothalamus or reticular activating system, it might help in our understanding of fatigue and sleep disorders in MS patients. Changes in genes for receptors for other transmitters such as dopamine and serotonin, which we have noted in data to be reported in a subsequent study, could also contribute to changes in mood, sleep and a sense of energy. In our earlier paper we also described down regulation of the gene for the gp130 receptor for leptin [34] which is not only involved in appetite control and food intake but also in energy metabolism. In addition, the widespread projections of the hypocretin neurons into other parts of the brain and spinal cord could influence other CNS signs and symptoms in MS including motor tone as well as sensory and autonomic function.

Th1 cytokines downregulated neurofilament heavy polypeptide as did M/M cytokines. M/M cytokines downregulated the gene for MAP 1A (-2-fold), while Th2 cytokines upregulated the gene for MAP 2 (3-fold). MAP 1 and MAP 2 are associated with mature neurons [188,189], although there is a report of expression of MAP 2 in oligodendrogliomas and glial precursor cells [190].

M/M cytokines down regulated the gene for synaptophysin, a synaptic vesicle glycoprotein [191] as well as for synaptotagmin 2, a calcium sensitive synaptic vesicle protein [192]. Th1 cytokines induced down regulation of the gene for activin receptor-like kinase 7 (ALK7), part of a family of receptor serine-threonine kinases (RSTK), which interact with TGF-β superfamily members. To date ALK7 has been associated with neurons of the cerebellum, hippocampus and brain stem nuclei [193]. Th1 cytokines upregulated the gene for activin receptor interacting protein 1, also called synaptic scaffolding molecule (S-SCAM) which resembles proteins that seem to be involved with the assembly of receptors and cell adhesion proteins at synaptic junctions [194,195]. M/M cytokines upregulated the gene for neuroligin 1 which interacts with beta-neurexins to help mediate synaptic development [196,197]. Th2 cytokines downregulated the genes for synapsin 3, a synaptic vesicular protein inhibited by Ca2+ [198], as well as semaphorin 3a; downregulation of this protein in MS or other diseases by cytokines would have major implications for pathogenesis and repair since semaphorin 3 inhibits growth cones as a chemorepellant and may be an important inhibitor of axonal regeneration and outgrowth. As a component of glial scars [199-202], semaphorin 3 could affect migration of neurons during development [203,204], as well as glial migration [205,206]. It may promote neuronal apoptosis [204] including that in retinal ganglion cells following optic nerve trauma [207], although interestingly it has also been reported to protect neurons from activated microglia [208]. In addition, production of semaphorin 3 by vascular endothelial cells inhibits vascular morphogenesis [209]. Th2 downregulated the gene for semaphorin 3a, which would favor axonal outgrowth.

Bassoon is a protein involved in synaptogenesis and in the structure of the presynaptic active zone; its deletion causes alterations in activation patterns and circuitry in the cortex [210-212]. The gene for bassoon was down regulated 5-fold by Th1 cytokines (Table 1).

VGF, upregulated 2 to 4-fold by all three cytokine mixtures, is a neuronal secreted peptide stimulated by neurotrophic factors [213,214] and is upregulated in neuronal development [215,216]. In addition to its role in neuronal development and plasticity, it also has neuroeondocrine properties and helps regulate energy metabolism [217].

It will be important to determine if these and other traditionally neuronal genes and their proteins are expressed in glial cells, as well as to determine the effect of cytokine mixtures on the genes and proteins of neurons since there are significant implications for neuronal/axonal damage as well as neuroprotection and inhibition of neuroprotective mechanisms due to changes in expression of these proteins.

### Retinal proteins

While there are no retinal ganglion cells or neurons in our cultures, we found changes in two proteins important in retinal diseases. Mutations in a human gene homologous to rat rds/peripherin 2 have been reported associated in an autosomal recessive inherited retinal disorder involving pigmented epithelium [218]. The normal function of rds is not known. Th2 cytokines upregulated the gene for rds/peripherin 2 which might thus slow degeneration of retinal cells in inflammatory or traumatic diseases if such Th2 cells were present in the retina. The gene for rhodopsin (mutated in retinitis pigmentosa 4, autosomal dominant) [219] is downregulated by M/M and Th2 cytokines, which raises the question of the role of cytokines in inducing retinal damage in inflammatory eye diseases.

### Cancer related proteins

It is not known if BRCA1 and DCC are expressed in glial cells and if so, their functions in glia are unknown. The gene for the BRCA1 was downregulated by all three cytokine mixtures. The rat homolog for the gene for the human protein DCC was downregulated by Th1 cytokines. M/M down regulated a gene for what is now still a theoretical protein, which is deleted in some gliomas. Th2 cytokines down regulated the gene for carcinoembryonic antigen. It is not known if expression of these gene and their products are affected by cytokines in tumor cells. BRCA1 is a tumor suppressor protein and is involved in DNA repair and cell growth. It is found in embryonic and adult neural stem cells and seems linked to proliferation [220]. DCC is a RAF kinase inhibitory protein (RKIF), previously called phosphtidylethanolamine binding protein, which seems to have tumor suppressor activity [221] and is deleted in several types of tumors, including some colorectal cancers in humans [222]. In the CNS it seems to play an important role in neuronal differentiation, migration and determination of cell fate. [222] DCC is the receptor for netrin-1 [223] and thus is important for axonal guidance [188,224,225]. As the netrin-1 receptor, it also affects oligodendroglial and OPC migration, in general inhibiting migration [226,227], which is the reverse of its usual effect on neuronal/axonal growth and migration. Netrins may also be involved in angiogenesis [228].

### Bone related proteins

We observed dramatic upregulation of the gene for BEST 5 by Th1 and M/M cytokines, 40-fold and 7-fold respectively. This factor has been reported to be upregulated in osteoblasts by both type I and type II interferons with a greater effect induced by type II IFN (IFN-γ). BEST 5 has been found in bone where its functions include modeling bone structure in response to physical stress [229]. To our knowledge, this is the first description of either expression or regulation of the gene for BEST 5 in cells of CNS origin.

M/M cytokines downregulated the gene for uterine sensitization-associated gene protein 1. (USAG1). The protein is a down regulator of the bone morphogenetic proteins (BMP), which are members of the TGF-β superfamily and found in the nervous system as well as being widely distributed in other organ systems. BMP are important in oligodendroglial, astrocyte and neuronal development [230,231].

## Conclusion

Gene expression studies in general and microarray studies in particular have some limitations. Post-transcriptional and post-translational changes are not detected and proteins may already be present and involved in a biologic process without requiring additional levels of protein and no upregulation of the gene for that protein might occur. However, as a screening technique to get an overview of proteins that may be important in a particular process as well as the complexities of the effect of a mixture of factors on a mixture of cells, we believe that this is a promising approach. In addition, microarray technology allows discovery of unexpected findings in complex experiments. Changes in gene expression detected with microarray technology need to be confirmed using more quantitative techniques and analysis of the effects of changes in gene regulation on protein expression are a required next step. Ultimately *in vitro *and *in vivo *experiments will be required to determine the biological consequences of the changes in gene expression described in these studies.

Comparison of the effects of the three cytokine mixtures reveals a pattern of changes unique for each mixture, most notably for the genes coding for growth/maturation factors, cell adhesion/ECM molecules and neuronal related proteins. Thus, Th1 cytokines uniquely regulated IGF-1 and insulin-like growth factor receptor 5, M/M cytokines regulated bFGF and FGF 5, while only Th2 cytokines regulated FGF 14, neuregulin 1, insulin-like growth factor II and PDGF receptor alpha. Th2 cytokines regulated expression of a large number of genes for adhesion/ECM molecules, while Th1 and M/M cytokines each uniquely regulated a larger number of neuronal related proteins than the Th2 cytokines. Further analysis of these patterns of changes in comparison to those seen in the CNS of patients with MS and EAE animals at various stages of disease and recovery promise to identify signaling networks that could be directed by pharmacologic strategies to promote neuroprotection and regeneration.

## Competing interests

Dr Lisak has served as a consultant to Teva Neuroscience as well as on the speakers' bureau for Teva Neuroscience. He has also served as a consultant to Genentech, Biogen/Idec, Serono and MediciNova. He has had research funding from Teva Neuroscience, Biogen/Idec, Serono, Berlex, Glaxo Smith Kline, BioMS, Abbott and Accorda. None of the authors hold stocks or shares in any pharmaceutical company or hold or are applying for any patents relating to the contents of the manuscript.

## Authors' contributions

RPL and JAB were involved in the conception, design, acquisition of data, analysis and interpretation of data, and the drafting of the manuscript. BB and LN performed the tissue culture experiments and BB performed the indirect immunofluorescence experiments. The molecular biological procedures were carried out under the supervision of SL and the biometric analysis was carried out by BY. The QRT-PCR was carried out by DS. All authors read and approved the final version.

## References

[B1] Raine CS (1994). The Dale E. McFarlin Memorial Lecture: the immunology of the multiple sclerosis lesion. Ann Neurol.

[B2] Brosnan CF, Cannella B, Battistini L, Raine CS (1995). Cytokine localization in multiple sclerosis lesions: correlation with adhesion molecule expression and reactive nitrogen species. Neurology.

[B3] Barnett MH, Prineas JW (2004). Relapsing and remitting multiple sclerosis: Pathology of the newly forming lesion. Ann Neurol.

[B4] Allen IV, McQuaid S, Mirakhur M, Nevin G (2001). Pathological abnormalities in the normal-appearing white matter in multiple sclerosis. Neurol Sci.

[B5] Lucchinetti C, Bruck W, Parisi J, Scheithauer B, Rodriguez M, Lassmann H (2000). Heterogeneity of multiple sclerosis lesions: implications for the pathogenesis of demyelination [see comments]. Ann Neurol.

[B6] Ferguson B, Matyszak MK, Esiri MM, Perry VH (1997). Axonal damage in acute multiple sclerosis lesions. Brain.

[B7] Trapp BD, Peterson J, Ransohoff RM, Rudick R, Mork S, Bo L (1998). Axonal transection in the lesions of multiple sclerosis [see comments]. N Engl J Med.

[B8] Peterson JW, Bo L, Mork S, Chang A, Trapp BD (2001). Transected neurites, apoptotic neurons, and reduced inflammation in cortical multiple sclerosis lesions. Ann Neurol.

[B9] Bo L, Vedeler CA, Nyland H, Trapp BD, Mork SJ (2003). Intracortical multiple sclerosis lesions are not associated with increased lymphocyte infiltration. Mult Scler.

[B10] McGeer PL, McGeer EG (1998). Glial cell reactions in neurodegenerative diseases: pathophysiology and therapeutic interventions. Alzheimer Dis Assoc Disord.

[B11] Akiyama H, Barger S, Barnum S, Bradt B, Bauer J, Cole GM, Cooper NR, Eikelenboom P, Emmerling M, Fiebich BL (2000). Inflammation and Alzheimer's disease. Neurobiol Aging.

[B12] McGeer EG, McGeer PL (1998). The importance of inflammatory mechanisms in Alzheimer disease. Exp Gerontol.

[B13] Beal MF (2003). Mitochondria, oxidative damage, and inflammation in Parkinson's disease. Ann N Y Acad Sci.

[B14] Bjartmar C, Wujek JR, Trapp BD (2003). Axonal loss in the pathology of MS: consequences for understanding the progressive phase of the disease. J Neurol Sci.

[B15] Trapp BD, Ransohoff R, Rudick R (1999). Axonal pathology in multiple sclerosis: relationship to neurologic disability. Curr Opin Neurol.

[B16] Yong VW (2004). Prospects for neuroprotection in multiple sclerosis. Front Biosci.

[B17] Hohlfeld R, Kerschensteiner M, Stadelmann C, Lassmann H, Wekerle H (2000). The neuroprotective effect of inflammation: implications for the therapy of multiple sclerosis. J Neuroimmunol.

[B18] Schwartz M, Moalem G, Leibowitz-Amit R, Cohen IR (1999). Innate and adaptive immune responses can be beneficial for CNS repair. Trends Neurosci.

[B19] Merrill J, Beneveniste E (1996). Cytokines in inflammatory brain lesions: helpful and harmful. Trends in Neurosci.

[B20] Kipnis J, Schwartz M (2002). Dual action of glatiramer acetate (Cop-1) in the treatment of CNS autoimmune and neurodegenerative disorders. Trends Mol Med.

[B21] Ure DR, Rodriguez M (2002). Polyreactive antibodies to glatiramer acetate promote myelin repair in murine model of demyelinating disease. Faseb J.

[B22] Lisak RP, Skundric D, Bealmear B, Ragheb S (1997). The role of cytokines in Schwann cell damage, protection, and repair. J Infect Dis.

[B23] Schwartz M (2000). Beneficial autoimmune T cells and posttraumatic neuroprotection. Ann N Y Acad Sci.

[B24] Hauben E, Agranov E, Gothilf A, Nevo U, Cohen A, Smirnov I, Steinman L, Schwartz M (2001). Posttraumatic therapeutic vaccination with modified myelin self-antigen prevents complete paralysis while avoiding autoimmune disease. J Clin Invest.

[B25] Angelov DN, Waibel S, Guntinas-Lichius O, Lenzen M, Neiss WF, Tomov TL, Yoles E, Kipnis J, Schori H, Reuter A (2003). Therapeutic vaccine for acute and chronic motor neuron diseases: implications for amyotrophic lateral sclerosis. Proc Natl Acad Sci USA.

[B26] Benner EJ, Mosley RL, Destache CJ, Lewis TB, Jackson-Lewis V, Gorantla S, Nemachek C, Green SR, Przedborski S, Gendelman HE (2004). Therapeutic immunization protects dopaminergic neurons in a mouse model of Parkinson's disease. Proc Natl Acad Sci USA.

[B27] Ge Y, Grossman RI, Udupa JK, Fulton J, Constantinescu CS, Gonzales-Scarano F, Babb JS, Mannon LJ, Kolson DL, Cohen JA (2000). Glatiramer acetate (Copaxone) treatment in relapsing-remitting MS: quantitative MR assessment. Neurology.

[B28] Filippi M, Rovaris M, Rocca MA, Sormani MP, Wolinsky JS, Comi G (2001). Glatiramer acetate reduces the proportion of new MS lesions evolving into "black holes". Neurology.

[B29] Sormani MP, Rovaris M, Valsasina P, Wolinsky JS, Comi G, Filippi M (2004). Measurement error of two different techniques for brain atrophy assessment in multiple sclerosis. Neurology.

[B30] Frank JA, Richert N, Bash C, Stone L, Calabresi PA, Lewis B, Stone R, Howard T, McFarland HF (2004). Interferon-beta-1b slows progression of atrophy in RRMS: Three-year follow-up in NAb- and NAb+ patients. Neurology.

[B31] Khan O, Shen Y, Caon C, Bao F, Ching W, Reznar M, Buccheister A, Hu J, Latif Z, Tselis A, Lisak R (2005). Axonal metabolic recovery and potential neuroprotective effect of glatiramer acetate in relapsing-remitting multiple sclerosis. Mult Scler.

[B32] Steinman L (2001). Gene microarrays and experimental demyelinating disease: a tool to enhance serendipity. Brain.

[B33] Martin R, Leppert D (2004). A plea for "Omics" research in complex diseases such as multiple sclerosis-a change of mind is needed. J Neurol Sci.

[B34] Lisak R, Benjamins J, Bealmear B, Yao B, Land S, Skundric DS (2006). Differential effects of Th1, monocyte/macrophage and Th2 cytokine mixtures on early gene expression for mmune-related molecules by central nervous system mixed glial cell cultures. Mult Scler.

[B35] McCarthy KD, de Vellis J (1980). Preparation of separate astroglial and oligodendroglial cell cultures from rat cerebral tissue. J Cell Biol.

[B36] Dyer CA, Benjamins JA (1988). Antibody to galactocerebroside alters organization of oligodendroglial membrane sheets in culture. J Neurosci.

[B37] Raff MC, Mirsky R, Fields KL, Lisak RP, Dorfman SH, Silberberg DH, Gregson NA, Leibowitz S, Kennedy MC (1978). Galactocerebroside is a specific cell-surface antigenic marker for oligodendrocytes in culture. Nature.

[B38] Ranscht B, Clapshaw PA, Price J, Noble M, Seifert W (1982). Development of oligodendrocytes and Schwann cells studied with a monoclonal antibody against galactocerebroside. Proc Natl Acad Sci USA.

[B39] Eisenbarth GS, Walsh FS, Nirenberg M (1979). Monoclonal antibody to a plasma membrane antigen of neurons. Proc Natl Acad Sci USA.

[B40] Dijkstra CD, Van Vliet E, Dopp EA, van der Lelij AA, Kraal G (1985). Marginal zone macrophages identified by a monoclonal antibody: characterization of immuno- and enzyme-histochemical properties and functional capacities. Immunology.

[B41] Mirsky R, Thompson EJ (1975). Thy 1 (theta) antigen on the surface of morphologically distinct brain cell types. Cell.

[B42] Pruss RM (1979). Thy-1 antigen on astrocytes in long-term cultures of rat central nervous system. Nature.

[B43] Sternberger LA, Harwell LW, Sternberger NH (1982). Neurotypy: regional individuality in rat brain detected by immunocytochemistry with monoclonal antibodies. Proc Natl Acad Sci USA.

[B44] Kim HJ, Ifergan I, Antel JP, Seguin R, Duddy M, Lapierre Y, Jalili F, Bar-Or A (2004). Type 2 monocyte and microglia differentiation mediated by glatiramer acetate therapy in patients with multiple sclerosis. J Immunol.

[B45] Liu Y, Teige I, Birnir B, Issazadeh-Navikas S (2006). Neuron-mediated generation of regulatory T cells from encephalitogenic T cells suppresses EAE. Nat Med.

[B46] Baecher-Allan C, Wolf E, Hafler DA (2005). Functional analysis of highly defined, FACS-isolated populations of human regulatory CD4+ CD25+ T cells. Clin Immunol.

[B47] Putheti P, Soderstrom M, Link H, Huang YM (2003). Effect of glatiramer acetate (Copaxone) on CD4+CD25high T regulatory cells and their IL-10 production in multiple sclerosis. J Neuroimmunol.

[B48] Selmaj K, Raine C, Farooq M, Norton W, Brosnan C (1991). Cytokine toxicity against oligodendrocytes. J Immunol.

[B49] Selmaj KW, Raine CS (1988). Tumor necrosis factor mediates myelin and oligodendrocyte damage in vitro. Ann Neurol.

[B50] Takahashi JL, Giuliani F, Power C, Imai Y, Yong VW (2003). Interleukin-1beta promotes oligodendrocyte death through glutamate excitotoxicity. Ann Neurol.

[B51] Beg AA, Baltimore D (1996). An essential role for NF-kappaB in preventing TNF-alpha-induced cell death [see comments]. Science.

[B52] Li C, Wong WH (2001). Model-based analysis of oligonucleotide arrays: expression index computation and outlier detection. Proc Natl Acad Sci USA.

[B53] Yao B, Rakhade SN, Li Q, Ahmed S, Krauss R, Draghici S, Loeb JA (2004). Accuracy of cDNA microarray methods to detect small gene expression changes induced by neuregulin on breast epithelial cells. BMC Bioinformatics.

[B54] Livak KJ, Schmittgen TD (2001). Analysis of relative gene expression data using real-time quantitative PCR and the 2(-Delta Delta C(T)) Method. Methods.

[B55] Benveniste EN, Merrill JE (1986). Stimulation of oligodendroglial proliferation and maturation by interleukin-2. Nature.

[B56] Patterson PH (1992). The emerging neuropoietic cytokine family: first CDF/LIF, CNTF and IL-6; next ONC, MGF, GCSF?. Curr Opin Neurobiol.

[B57] Kerschensteiner M, Gallmeier E, Behrens L, Leal VV, Misgeld T, Klinkert WE, Kolbeck R, Hoppe E, Oropeza-Wekerle RL, Bartke I (1999). Activated human T cells, B cells, and monocytes produce brain-derived neurotrophic factor in vitro and in inflammatory brain lesions: a neuroprotective role of inflammation?. J Exp Med.

[B58] Ziemssen T, Kumpfel T, Klinkert WE, Neuhaus O, Hohlfeld R (2002). Glatiramer acetate-specific T-helper 1- and 2-type cell lines produce BDNF: implications for multiple sclerosis therapy. Brain-derived neurotrophic factor. Brain.

[B59] Chen M, Valenzuela RM, Dhib-Jalbut S (2003). Glatiramer acetate-reactive T cells produce brain-derived neurotrophic factor. J Neurol Sci.

[B60] Lindholm D, Heumann R, Meyer M, Thoenen H (1987). Interleukin-1 regulates synthesis of nerve growth factor in non-neuronal cells of rat sciatic nerve. Nature.

[B61] Lindholm D, Hengerer B, Zafra F, Thoenen H (1990). Transforming growth factor-beta 1 stimulates expression of nerve growth factor in the rat CNS. Neuroreport.

[B62] Bedard A, Tremblay P, Chernomoretz A, Vallieres L (2007). Identification of genes preferentially expressed by microglia and upregulated during cuprizone-induced inflammation. Glia.

[B63] Cua DJ, Sherlock J, Chen Y, Murphy CA, Joyce B, Seymour B, Lucian L, To W, Kwan S, Churakova T (2003). Interleukin-23 rather than interleukin-12 is the critical cytokine for autoimmune inflammation of the brain. Nature.

[B64] Sutton C, Brereton C, Keogh B, Mills KH, Lavelle EC (2006). A crucial role for interleukin (IL)-1 in the induction of IL-17-producing T cells that mediate autoimmune encephalomyelitis. J Exp Med.

[B65] Batten M, Li J, Yi S, Kljavin NM, Danilenko DM, Lucas S, Lee J, de Sauvage FJ, Ghilardi N (2006). Interleukin 27 limits autoimmune encephalomyelitis by suppressing the development of interleukin 17-producing T cells. Nat Immunol.

[B66] Qian Y, Liu C, Hartupee J, Altuntas CZ, Gulen MF, Jane-Wit D, Xiao J, Lu Y, Giltiay N, Liu J (2007). The adaptor Act1 is required for interleukin 17-dependent signaling associated with autoimmune and inflammatory disease. Nat Immunol.

[B67] Chen Y, Langrish CL, McKenzie B, Joyce-Shaikh B, Stumhofer JS, McClanahan T, Blumenschein W, Churakovsa T, Low J, Presta L (2006). Anti-IL-23 therapy inhibits multiple inflammatory pathways and ameliorates autoimmune encephalomyelitis. J Clin Invest.

[B68] Touil T, Fitzgerald D, Zhang GX, Rostami AM, Gran B (2006). Pathophysiology of interleukin-23 in experimental autoimmune encephalomyelitis. Drug News Perspect.

[B69] Lock C, Hermans G, Pedotti R, Brendolan A, Schadt E, Garren H, Langer-Gould A, Strober S, Cannella B, Allard J (2002). Gene-microarray analysis of multiple sclerosis lesions yields new targets validated in autoimmune encephalomyelitis. Nat Med.

[B70] Matusevicius D, Kivisakk P, He B, Kostulas N, Ozenci V, Fredrikson S, Link H (1999). Interleukin-17 mRNA expression in blood and CSF mononuclear cells is augmented in multiple sclerosis. Mult Scler.

[B71] Ishizu T, Osoegawa M, Mei FJ, Kikuchi H, Tanaka M, Takakura Y, Minohara M, Murai H, Mihara F, Taniwaki T, Kira J (2005). Intrathecal activation of the IL-17/IL-8 axis in opticospinal multiple sclerosis. Brain.

[B72] Wilson NJ, Boniface K, Chan JR, McKenzie BS, Blumenschein WM, Mattson JD, Basham B, Smith K, Chen T, Morel F (2007). Development, cytokine profile and function of human interleukin 17-producing helper T cells. Nat Immunol.

[B73] Witowski J, Ksiazek K, Jorres A (2004). Interleukin-17: a mediator of inflammatory responses. Cell Mol Life Sci.

[B74] Kolls JK, Linden A (2004). Interleukin-17 family members and inflammation. Immunity.

[B75] Friedman WJ, Black IB, Kaplan DR (1998). Distribution of the neurotrophins brain-derived neurotrophic factor, neurotrophin-3, and neurotrophin-4/5 in the postnatal rat brain: an immunocytochemical study. Neuroscience.

[B76] Hyman C, Juhasz M, Jackson C, Wright P, Ip NY, Lindsay RM (1994). Overlapping and distinct actions of the neurotrophins BDNF, NT-3, and NT-4/5 on cultured dopaminergic and GABAergic neurons of the ventral mesencephalon. J Neurosci.

[B77] Friedman B, Kleinfeld D, Ip NY, Verge VM, Moulton R, Boland P, Zlotchenko E, Lindsay RM, Liu L (1995). BDNF and NT-4/5 exert neurotrophic influences on injured adult spinal motor neurons. J Neurosci.

[B78] McTigue DM, Horner PJ, Stokes BT, Gage FH (1998). Neurotrophin-3 and brain-derived neurotrophic factor induce oligodendrocyte proliferation and myelination of regenerating axons in the contused adult rat spinal cord. J Neurosci.

[B79] Cai D, Shen Y, De Bellard M, Tang S, Filbin MT (1999). Prior exposure to neurotrophins blocks inhibition of axonal regeneration by MAG and myelin via a cAMP-dependent mechanism. Neuron.

[B80] Dougherty KD, Dreyfus CF, Black IB (2000). Brain-derived neurotrophic factor in astrocytes, oligodendrocytes, and microglia/macrophages after spinal cord injury. Neurobiol Dis.

[B81] Barouch R, Schwartz M (2002). Autoreactive T cells induce neurotrophin production by immune and neural cells in injured rat optic nerve: implications for protective autoimmunity. Faseb J.

[B82] Stadelmann C, Kerschensteiner M, Misgeld T, Bruck W, Hohlfeld R, Lassmann H (2002). BDNF and gp145trkB in multiple sclerosis brain lesions: neuroprotective interactions between immune and neuronal cells?. Brain.

[B83] Mycko MP, Papoian R, Boschert U, Raine CS, Selmaj KW (2003). cDNA microarray analysis in multiple sclerosis lesions: detection of genes associated with disease activity. Brain.

[B84] Barres BA, Raff MC, Gaese F, Bartke I, Dechant G, Barde YA (1994). A crucial role for neurotrophin-3 in oligodendrocyte development. Nature.

[B85] Tobias CA, Shumsky JS, Shibata M, Tuszynski MH, Fischer I, Tessler A, Murray M (2003). Delayed grafting of BDNF and NT-3 producing fibroblasts into the injured spinal cord stimulates sprouting, partially rescues axotomized red nucleus neurons from loss and atrophy, and provides limited regeneration. Exp Neurol.

[B86] Bredesen DE, Rabizadeh S (1997). p75NTR and apoptosis: Trk-dependent and Trk-independent effects. Trends Neurosci.

[B87] Casaccia-Bonnefil P, Gu C, Chao MV (1999). Neurotrophins in cell survival/death decisions. Adv Exp Med Biol.

[B88] Wang KC, Kim JA, Sivasankaran R, Segal R, He Z (2002). P75 interacts with the Nogo receptor as a co-receptor for Nogo, MAG and OMgp. Nature.

[B89] Chalazonitis A, Kessler JA, Twardzik DR, Morrison RS (1992). Transforming growth factor alpha, but not epidermal growth factor, promotes the survival of sensory neurons in vitro. J Neurosci.

[B90] Li X, Sankrithi N, Davis FC (2002). Transforming growth factor-alpha is expressed in astrocytes of the suprachiasmatic nucleus in hamster: role of glial cells in circadian clocks. Neuroreport.

[B91] Zelenaia O, Schlag BD, Gochenauer GE, Ganel R, Song W, Beesley JS, Grinspan JB, Rothstein JD, Robinson MB (2000). Epidermal growth factor receptor agonists increase expression of glutamate transporter GLT-1 in astrocytes through pathways dependent on phosphatidylinositol 3-kinase and transcription factor NF-kappaB. Mol Pharmacol.

[B92] Yoshida T, Satoh M, Nakagaito Y, Kuno H, Takeuchi M (1993). Cytokines affecting survival and differentiation of an astrocyte progenitor cell line. Brain Res Dev Brain Res.

[B93] Namba H, Takei N, Nawa H (2003). Transforming growth factor-alpha changes firing properties of developing neocortical GABAergic neurons by down-regulation of voltage-gated potassium currents. Neuroscience.

[B94] Tang P, Steck PA, Yung WK (1997). The autocrine loop of TGF-alpha/EGFR and brain tumors. J Neurooncol.

[B95] Ma L, Gauville C, Berthois Y, Degeorges A, Millot G, Martin PM, Calvo F (1998). Role of epidermal-growth-factor receptor in tumor progression in transformed human mammary epithelial cells. Int J Cancer.

[B96] Matsuura H, Sakaue M, Subbaramaiah K, Kamitani H, Eling TE, Dannenberg AJ, Tanabe T, Inoue H, Arata J, Jetten AM (1999). Regulation of cyclooxygenase-2 by interferon gamma and transforming growth factor alpha in normal human epidermal keratinocytes and squamous carcinoma cells. Role of mitogen-activated protein kinases. J Biol Chem.

[B97] Almazan G, Honegger P, Matthieu JM, Guentert-Lauber B (1985). Epidermal growth factor and bovine growth hormone stimulate differentiation and myelination of brain cell aggregates in culture. Brain Res.

[B98] Chandran S, Svendsen C, Compston A, Scolding N (1998). Regional potential for oligodendrocyte generation in the rodent embryonic spinal cord following exposure to EGF and FGF-2. Glia.

[B99] Hammang JP, Archer DR, Duncan ID (1997). Myelination following transplantation of EGF-responsive neural stem cells into a myelin-deficient environment. Exp Neurol.

[B100] Kuhn HG, Winkler J, Kempermann G, Thal LJ, Gage FH (1997). Epidermal growth factor and fibroblast growth factor-2 have different effects on neural progenitors in the adult rat brain. J Neurosci.

[B101] Lie DC, Song H, Colamarino SA, Ming GL, Gage FH (2004). Neurogenesis in the adult brain: new strategies for central nervous system diseases. Annu Rev Pharmacol Toxicol.

[B102] Messersmith DJ, Murtie JC, Le TQ, Frost EE, Armstrong RC (2000). Fibroblast growth factor 2 (FGF2) and FGF receptor expression in an experimental demyelinating disease with extensive remyelination. J Neurosci Res.

[B103] Ruffini F, Furlan R, Poliani PL, Brambilla E, Marconi PC, Bergami A, Desina G, Glorioso JC, Comi G, Martino G (2001). Fibroblast growth factor-II gene therapy reverts the clinical course and the pathological signs of chronic experimental autoimmune encephalomyelitis in C57BL/6 mice. Gene Ther.

[B104] Bogler O, Wren D, Barnett SC, Land H, Noble M (1990). Cooperation between two growth factors promotes extended self-renewal and inhibits differentiation of oligodendrocyte-type-2 astrocyte (O-2A) progenitor cells. Proc Natl Acad Sci USA.

[B105] Armstrong RC, Le TQ, Frost EE, Borke RC, Vana AC (2002). Absence of fibroblast growth factor 2 promotes oligodendroglial repopulation of demyelinated white matter. J Neurosci.

[B106] Fok-Seang J, DiProspero NA, Meiners S, Muir E, Fawcett JW (1998). Cytokine-induced changes in the ability of astrocytes to support migration of oligodendrocyte precursors and axon growth. Eur J Neurosci.

[B107] Copelman CA, Cuzner ML, Groome N, Diemel LT (2000). Temporal analysis of growth factor mRNA expression in myelinating rat brain aggregate cultures: increments in CNTF, FGF-2, IGF-I, and PDGF-AA mRNA are induced by antibody-mediated demyelination. Glia.

[B108] Maric D, Maric I, Chang YH, Barker JL (2003). Prospective cell sorting of embryonic rat neural stem cells and neuronal and glial progenitors reveals selective effects of basic fibroblast growth factor and epidermal growth factor on self-renewal and differentiation. J Neurosci.

[B109] Reimers D, Lopez-Toledano MA, Mason I, Cuevas P, Redondo C, Herranz AS, Lobo MV, Bazan E (2001). Developmental expression of fibroblast growth factor (FGF) receptors in neural stem cell progeny. Modulation of neuronal and glial lineages by basic FGF treatment. Neurol Res.

[B110] Barres BA, Hart IK, Coles HS, Burne JF, Voyvodic JT, Richardson WD, Raff MC (1992). Cell death in the oligodendrocyte lineage. J Neurobiol.

[B111] Wolswijk G, Noble M (1992). Cooperation between PDGF and FGF converts slowly dividing O-2Aadult progenitor cells to rapidly dividing cells with characteristics of O-2Aperinatal progenitor cells. J Cell Biol.

[B112] Hinks GL, Franklin RJ (1999). Distinctive patterns of PDGF-A, FGF-2, IGF-I, and TGF-beta1 gene expression during remyelination of experimentally-induced spinal cord demyelination. Mol Cell Neurosci.

[B113] Robinson S, Tani M, Strieter RM, Ransohoff RM, Miller RH (1998). The chemokine growth-regulated oncogene-alpha promotes spinal cord oligodendrocyte precursor proliferation. J Neurosci.

[B114] Wu Q, Miller RH, Ransohoff RM, Robinson S, Bu J, Nishiyama A (2000). Elevated levels of the chemokine GRO-1 correlate with elevated oligodendrocyte progenitor proliferation in the jimpy mutant. J Neurosci.

[B115] Cheng HL, Feldman EL (1997). Insulin-like growth factor-I (IGF-I) and IGF binding protein-5 in Schwann cell differentiation. J Cell Physiol.

[B116] Deadwyler GD, Pouly S, Antel JP, Devries GH (2000). Neuregulins and erbB receptor expression in adult human oligodendrocytes [In Process Citation]. Glia.

[B117] Buonanno A, Fischbach GD (2001). Neuregulin and ErbB receptor signaling pathways in the nervous system. Curr Opin Neurobiol.

[B118] Fernandez PA, Tang DG, Cheng L, Prochiantz A, Mudge AW, Raff MC (2000). Evidence that axon-derived neuregulin promotes oligodendrocyte survival in the developing rat optic nerve. Neuron.

[B119] Kim JY, Sun Q, Oglesbee M, Yoon SO (2003). The role of ErbB2 signaling in the onset of terminal differentiation of oligodendrocytes in vivo. J Neurosci.

[B120] Michailov GV, Sereda MW, Brinkmann BG, Fischer TM, Haug B, Birchmeier C, Role L, Lai C, Schwab MH, Nave KA (2004). Axonal neuregulin-1 regulates myelin sheath thickness. Science.

[B121] Esper RM, Loeb JA (2004). Rapid axoglial signaling mediated by neuregulin and neurotrophic factors. J Neurosci.

[B122] Viehover A, Miller RH, Park SK, Fischbach G, Vartanian T (2001). Neuregulin: an oligodendrocyte growth factor absent in active multiple sclerosis lesions. Dev Neurosci.

[B123] Cannella B, Pitt D, Marchionni M, Raine CS (1999). Neuregulin and erbB receptor expression in normal and diseased human white matter. J Neuroimmunol.

[B124] Baron-Van Evercooren A, Olichon-Berthe C, Kowalski A, Visciano G, Van Obberghen E (1991). Expression of IGF-I and insulin receptor genes in the rat central nervous system: a developmental, regional, and cellular analysis. J Neurosci Res.

[B125] Barres BA, Schmid R, Sendnter M, Raff MC (1993). Multiple extracellular signals are required for long-term oligodendrocyte survival. Development.

[B126] Kermer P, Klocker N, Labes M, Bahr M (2000). Insulin-like growth factor-I protects axotomized rat retinal ganglion cells from secondary death via PI3-K-dependent Akt phosphorylation and inhibition of caspase-3 In vivo. J Neurosci.

[B127] McMorris FA, Dubois-Dalcq M (1988). Insulin-like growth factor I promotes cell proliferation and oligodendroglial commitment in rat glial progenitor cells developing in vitro. J Neurosci Res.

[B128] Yao DL, Liu X, Hudson LD, Webster HD (1996). Insulin-like growth factor-I given subcutaneously reduces clinical deficits, decreases lesion severity and upregulates synthesis of myelin proteins in experimental autoimmune encephalomyelitis. Life Sci.

[B129] Lovett-Racke AE, Bittner P, Cross AH, Carlino JA, Racke MK (1998). Regulation of experimental autoimmune encephalomyelitis with insulin-like growth factor (IGF-1) and IGF-1/IGF-binding protein-3 complex (IGF-1/IGFBP3). J Clin Invest.

[B130] Cannella B, Pitt D, Capello E, Raine CS (2000). Insulin-like growth factor-1 fails to enhance central nervous system myelin repair during autoimmune demyelination. Am J Pathol.

[B131] Genoud S, Maricic I, Kumar V, Gage FH (2005). Targeted expression of IGF-1 in the central nervous system fails to protect mice from experimental autoimmune encephalomyelitis. J Neuroimmunol.

[B132] Hammarberg H, Risling M, Hokfelt T, Cullheim S, Piehl F (1998). Expression of insulin-like growth factors and corresponding binding proteins (IGFBP 1–6) in rat spinal cord and peripheral nerve after axonal injuries. J Comp Neurol.

[B133] Nicholas RS, Stevens S, Wing MG, Compston DA (2002). Microglia-derived IGF-2 prevents TNFalpha induced death of mature oligodendrocytes in vitro. J Neuroimmunol.

[B134] Hobson MI, Green CJ, Terenghi G (2000). VEGF enhances intraneural angiogenesis and improves nerve regeneration after axotomy. J Anat.

[B135] Kirk SL, Karlik SJ (2003). VEGF and vascular changes in chronic neuroinflammation. J Autoimmun.

[B136] Jin K, Zhu Y, Sun Y, Mao XO, Xie L, Greenberg DA (2002). Vascular endothelial growth factor (VEGF) stimulates neurogenesis in vitro and in vivo. Proc Natl Acad Sci USA.

[B137] Maurer MH, Tripps WK, Feldmann RE, Kuschinsky W (2003). Expression of vascular endothelial growth factor and its receptors in rat neural stem cells. Neurosci Lett.

[B138] Rosenstein JM, Mani N, Khaibullina A, Krum JM (2003). Neurotrophic effects of vascular endothelial growth factor on organotypic cortical explants and primary cortical neurons. J Neurosci.

[B139] Proescholdt MA, Jacobson S, Tresser N, Oldfield EH, Merrill MJ (2002). Vascular endothelial growth factor is expressed in multiple sclerosis plaques and can induce inflammatory lesions in experimental allergic encephalomyelitis rats. J Neuropathol Exp Neurol.

[B140] Proescholdt MA, Heiss JD, Walbridge S, Muhlhauser J, Capogrossi MC, Oldfield EH, Merrill MJ (1999). Vascular endothelial growth factor (VEGF) modulates vascular permeability and inflammation in rat brain. J Neuropathol Exp Neurol.

[B141] Gregg C, Shikar V, Larsen P, Mak G, Chojnacki A, Yong VW, Weiss S (2007). White matter plasticity and enhanced remyelination in the maternal CNS. J Neurosci.

[B142] Bandtlow CE, Zimmermann DR (2000). Proteoglycans in the developing brain: new conceptual insights for old proteins. Physiol Rev.

[B143] Hartmann U, Maurer P (2001). Proteoglycans in the nervous system – the quest for functional roles in vivo. Matrix Biol.

[B144] Bansal R, Kumar M, Murray K, Pfeiffer SE (1996). Developmental and FGF-2-mediated regulation of syndecans (1–4) and glypican in oligodendrocytes. Mol Cell Neurosci.

[B145] Tucker RP, Hammarback JA, Jenrath DA, Mackie EJ, Xu Y (1993). Tenascin expression in the mouse: in situ localization and induction in vitro by bFGF. J Cell Sci.

[B146] Grumet M, Friedlander DR, Sakurai T (1996). Functions of brain chondroitin sulfate proteoglycans during developments: interactions with adhesion molecules. Perspect Dev Neurobiol.

[B147] Bachmann M, Conscience JF, Probstmeier R, Carbonetto S, Schachner M (1995). Recognition molecules myelin-associated glycoprotein and tenascin-C inhibit integrin-mediated adhesion of neural cells to collagen. J Neurosci Res.

[B148] Faissner A (1997). The tenascin gene family in axon growth and guidance. Cell Tissue Res.

[B149] Fawcett JW, Asher RA (1999). The glial scar and central nervous system repair. Brain Res Bull.

[B150] Kiernan BW, Gotz B, Faissner A, ffrench-Constant C (1996). Tenascin-C inhibits oligodendrocyte precursor cell migration by both adhesion-dependent and adhesion-independent mechanisms. Mol Cell Neurosci.

[B151] Kakinuma Y, Saito F, Osawa S, Miura M (2004). A mechanism of impaired mobility of oligodendrocyte progenitor cells by tenascin C through modification of wnt signaling. FEBS Lett.

[B152] Joester A, Faissner A (2001). The structure and function of tenascins in the nervous system. Matrix Biol.

[B153] Tucker RP (2001). Abnormal neural crest cell migration after the in vivo knockdown of tenascin-C expression with morpholino antisense oligonucleotides. Dev Dyn.

[B154] Gutowski NJ, Newcombe J, Cuzner ML (1999). Tenascin-R and C in multiple sclerosis lesions: relevance to extracellular matrix remodelling. Neuropathol Appl Neurobiol.

[B155] Hughes-Benzie RM, Hunter AG, Allanson JE, Mackenzie AE (1992). Simpson-Golabi-Behmel syndrome associated with renal dysplasia and embryonal tumor: localization of the gene to Xqcen-q21. Am J Med Genet.

[B156] Verloes A, Massart B, Dehalleux I, Langhendries JP, Koulischer L (1995). Clinical overlap of Beckwith-Wiedemann, Perlman and Simpson-Golabi-Behmel syndromes: a diagnostic pitfall. Clin Genet.

[B157] Weksberg R, Shen DR, Fei YL, Song QL, Squire J (1993). Disruption of insulin-like growth factor 2 imprinting in Beckwith-Wiedemann syndrome. Nat Genet.

[B158] Grimpe B, Pressman Y, Lupa MD, Horn KP, Bunge MB, Silver J (2005). The role of proteoglycans in Schwann cell/astrocyte interactions and in regeneration failure at PNS/CNS interfaces. Mol Cell Neurosci.

[B159] Fouad K, Schnell L, Bunge MB, Schwab ME, Liebscher T, Pearse DD (2005). Combining Schwann cell bridges and olfactory-ensheathing glia grafts with chondroitinase promotes locomotor recovery after complete transection of the spinal cord. J Neurosci.

[B160] Gil OD, Zanazzi G, Struyk AF, Salzer JL (1998). Neurotrimin mediates bifunctional effects on neurite outgrowth via homophilic and heterophilic interactions. J Neurosci.

[B161] Struyk AF, Canoll PD, Wolfgang MJ, Rosen CL, D'Eustachio P, Salzer JL (1995). Cloning of neurotrimin defines a new subfamily of differentially expressed neural cell adhesion molecules. J Neurosci.

[B162] Zeng L, Zhang C, Xu J, Ye X, Wu Q, Dai J, Ji C, Gu S, Xie Y, Mao Y (2002). A novel splice variant of the cell adhesion molecule contactin 4 (CNTN4) is mainly expressed in human brain. J Hum Genet.

[B163] Rios JC, Melendez-Vasquez CV, Einheber S, Lustig M, Grumet M, Hemperly J, Peles E, Salzer JL (2000). Contactin-associated protein (Caspr) and contactin form a complex that is targeted to the paranodal junctions during myelination. J Neurosci.

[B164] Einheber S, Zanazzi G, Ching W, Scherer S, Milner TA, Peles E, Salzer JL (1997). The axonal membrane protein Caspr, a homologue of neurexin IV, is a component of the septate-like paranodal junctions that assemble during myelination. J Cell Biol.

[B165] Wolswijk G, Balesar R (2003). Changes in the expression and localization of the paranodal protein Caspr on axons in chronic multiple sclerosis. Brain.

[B166] Fernandez T, Morgan T, Davis N, Klin A, Morris A, Farhi A, Lifton RP, State MW (2004). Disruption of contactin 4 (CNTN4) results in developmental delay and other features of 3p deletion syndrome. Am J Hum Genet.

[B167] Usami Y, Chiba H, Nakayama F, Ueda J, Matsuda Y, Sawada N, Komori T, Ito A, Yokozaki H (2006). Reduced expression of claudin-7 correlates with invasion and metastasis in squamous cell carcinoma of the esophagus. Hum Pathol.

[B168] Gonzalez-Mariscal L, Betanzos A, Nava P, Jaramillo BE (2003). Tight junction proteins. Prog Biophys Mol Biol.

[B169] Wolburg H, Wolburg-Buchholz K, Kraus J, Rascher-Eggstein G, Liebner S, Hamm S, Duffner F, Grote EH, Risau W, Engelhardt B (2003). Localization of claudin-3 in tight junctions of the blood-brain barrier is selectively lost during experimental autoimmune encephalomyelitis and human glioblastoma multiforme. Acta Neuropathol.

[B170] Wolburg H, Wolburg-Buchholz K, Liebner S, Engelhardt B (2001). Claudin-1, claudin-2 and claudin-11 are present in tight junctions of choroid plexus epithelium of the mouse. Neurosci Lett.

[B171] Gow A, Southwood CM, Li JS, Pariali M, Riordan GP, Brodie SE, Danias J, Bronstein JM, Kachar B, Lazzarini RA (1999). CNS myelin and sertoli cell tight junction strands are absent in Osp/claudin-11 null mice. Cell.

[B172] Bronstein JM, Tiwari-Woodruff S, Buznikov AG, Stevens DB (2000). Involvement of OSP/claudin-11 in oligodendrocyte membrane interactions: role in biology and disease. J Neurosci Res.

[B173] Evans WH, Martin PE (2002). Gap junctions: structure and function (Review). Mol Membr Biol.

[B174] Goodenough DA, Goliger JA, Paul DL (1996). Connexins, connexons, and intercellular communication. Annu Rev Biochem.

[B175] Menichella DM, Goodenough DA, Sirkowski E, Scherer SS, Paul DL (2003). Connexins are critical for normal myelination in the CNS. J Neurosci.

[B176] Xia AP, Kikuchi T, Minowa O, Katori Y, Oshima T, Noda T, Ikeda K (2002). Late-onset hearing loss in a mouse model of DFN3 non-syndromic deafness: morphologic and immunohistochemical analyses. Hear Res.

[B177] Chang EH, Van Camp G, Smith RJ (2003). The role of connexins in human disease. Ear Hear.

[B178] Lopez-Bigas N, Olive M, Rabionet R, Ben-David O, Martinez-Matos JA, Bravo O, Banchs I, Volpini V, Gasparini P, Avraham KB (2001). Connexin 31 (GJB3) is expressed in the peripheral and auditory nerves and causes neuropathy and hearing impairment. Hum Mol Genet.

[B179] Benveniste EN, Herman PK, Whitaker JN (1987). Myelin basic protein-specific RNA levels in interleukin-2-stimulated oligodendrocytes. J Neurochem.

[B180] Scammell TE (2003). The neurobiology, diagnosis, and treatment of narcolepsy. Ann Neurol.

[B181] Ripley B, Overeem S, Fujiki N, Nevsimalova S, Uchino M, Yesavage J, Di Monte D, Dohi K, Melberg A, Lammers GJ (2001). CSF hypocretin/orexin levels in narcolepsy and other neurological conditions. Neurology.

[B182] Thannickal TC, Siegel JM, Nienhuis R, Moore RY (2003). Pattern of hypocretin (orexin) soma and axon loss, and gliosis, in human narcolepsy. Brain Pathol.

[B183] Baumann CR, Bassetti CL (2005). Hypocretins (orexins) and sleep-wake disorders. Lancet Neurol.

[B184] Nishino S, Ripley B, Overeem S, Nevsimalova S, Lammers GJ, Vankova J, Okun M, Rogers W, Brooks S, Mignot E (2001). Low cerebrospinal fluid hypocretin (Orexin) and altered energy homeostasis in human narcolepsy. Ann Neurol.

[B185] Kunii K, Yamanaka A, Nambu T, Matsuzaki I, Goto K, Sakurai T (1999). Orexins/hypocretins regulate drinking behaviour. Brain Res.

[B186] van den Pol AN (1999). Hypothalamic hypocretin (orexin): robust innervation of the spinal cord. J Neurosci.

[B187] Peyron C, Tighe DK, van den Pol AN, de Lecea L, Heller HC, Sutcliffe JG, Kilduff TS (1998). Neurons containing hypocretin (orexin) project to multiple neuronal systems. J Neurosci.

[B188] Barallobre MJ, Pascual M, Del Rio JA, Soriano E (2005). The Netrin family of guidance factors: emphasis on Netrin-1 signalling. Brain Res Brain Res Rev.

[B189] Milosevic A, Goldman JE (2002). Progenitors in the postnatal cerebellar white matter are antigenically heterogeneous. J Comp Neurol.

[B190] Blumcke I, Becker AJ, Normann S, Hans V, Riederer BM, Krajewski S, Wiestler OD, Reifenberger G (2001). Distinct expression pattern of microtubule-associated protein-2 in human oligodendrogliomas and glial precursor cells. J Neuropathol Exp Neurol.

[B191] Stottmann RW, Rivas RJ (1998). Distribution of TAG-1 and synaptophysin in the developing cerebellar cortex: relationship to Purkinje cell dendritic development. J Comp Neurol.

[B192] Brose N, Petrenko AG, Sudhof TC, Jahn R (1992). Synaptotagmin: a calcium sensor on the synaptic vesicle surface. Science.

[B193] Ryden M, Imamura T, Jornvall H, Belluardo N, Neveu I, Trupp M, Okadome T, ten Dijke P, Ibanez CF (1996). A novel type I receptor serine-threonine kinase predominantly expressed in the adult central nervous system. J Biol Chem.

[B194] Hirao K, Hata Y, Ide N, Takeuchi M, Irie M, Yao I, Deguchi M, Toyoda A, Sudhof TC, Takai Y (1998). A novel multiple PDZ domain-containing molecule interacting with N-methyl-D-aspartate receptors and neuronal cell adhesion proteins. J Biol Chem.

[B195] Hirao K, Hata Y, Yao I, Deguchi M, Kawabe H, Mizoguchi A, Takai Y (2000). Three isoforms of synaptic scaffolding molecule and their characterization. Multimerization between the isoforms and their interaction with N-methyl-D-aspartate receptors and SAP90/PSD-95-associated protein. J Biol Chem.

[B196] Scheiffele P, Fan J, Choih J, Fetter R, Serafini T (2000). Neuroligin expressed in nonneuronal cells triggers presynaptic development in contacting axons. Cell.

[B197] Dean C, Scholl FG, Choih J, DeMaria S, Berger J, Isacoff E, Scheiffele P (2003). Neurexin mediates the assembly of presynaptic terminals. Nat Neurosci.

[B198] Hosaka M, Sudhof TC (1998). Synapsin III, a novel synapsin with an unusual regulation by Ca2+. J Biol Chem.

[B199] Pasterkamp RJ, Giger RJ, Ruitenberg MJ, Holtmaat AJ, De Wit J, De Winter F, Verhaagen J (1999). Expression of the gene encoding the chemorepellent semaphorin III is induced in the fibroblast component of neural scar tissue formed following injuries of adult but not neonatal CNS. Mol Cell Neurosci.

[B200] Dent EW, Barnes AM, Tang F, Kalil K (2004). Netrin-1 and semaphorin 3A promote or inhibit cortical axon branching, respectively, by reorganization of the cytoskeleton. J Neurosci.

[B201] Nakamura F, Kalb RG, Strittmatter SM (2000). Molecular basis of semaphorin-mediated axon guidance. J Neurobiol.

[B202] Mark MD, Lohrum M, Puschel AW (1997). Patterning neuronal connections by chemorepulsion: the semaphorins. Cell Tissue Res.

[B203] Tamamaki N, Fujimori K, Nojyo Y, Kaneko T, Takauji R (2003). Evidence that Sema3A and Sema3F regulate the migration of GABAergic neurons in the developing neocortex. J Comp Neurol.

[B204] Bagnard D, Vaillant C, Khuth ST, Dufay N, Lohrum M, Puschel AW, Belin MF, Bolz J, Thomasset N (2001). Semaphorin 3A-vascular endothelial growth factor-165 balance mediates migration and apoptosis of neural progenitor cells by the recruitment of shared receptor. J Neurosci.

[B205] Tsai HH, Miller RH (2002). Glial cell migration directed by axon guidance cues. Trends Neurosci.

[B206] Spassky N, de Castro F, Le Bras B, Heydon K, Queraud-LeSaux F, Bloch-Gallego E, Chedotal A, Zalc B, Thomas JL (2002). Directional guidance of oligodendroglial migration by class 3 semaphorins and netrin-1. J Neurosci.

[B207] Shirvan A, Kimron M, Holdengreber V, Ziv I, Ben-Shaul Y, Melamed S, Melamed E, Barzilai A, Solomon AS (2002). Anti-semaphorin 3A antibodies rescue retinal ganglion cells from cell death following optic nerve axotomy. J Biol Chem.

[B208] Majed HH, Chandran S, Niclou SP, Nicholas RS, Wilkins A, Wing MG, Rhodes KE, Spillantini MG, Compston A (2006). A novel role for Sema3A in neuroprotection from injury mediated by activated microglia. J Neurosci.

[B209] Serini G, Valdembri D, Zanivan S, Morterra G, Burkhardt C, Caccavari F, Zammataro L, Primo L, Tamagnone L, Logan M (2003). Class 3 semaphorins control vascular morphogenesis by inhibiting integrin function. Nature.

[B210] Dresbach T, Hempelmann A, Spilker C, tom Dieck S, Altrock WD, Zuschratter W, Garner CC, Gundelfinger ED (2003). Functional regions of the presynaptic cytomatrix protein bassoon: significance for synaptic targeting and cytomatrix anchoring. Mol Cell Neurosci.

[B211] tom Dieck S, Sanmarti-Vila L, Langnaese K, Richter K, Kindler S, Soyke A, Wex H, Smalla KH, Kampf U, Franzer JT (1998). Bassoon, a novel zinc-finger CAG/glutamine-repeat protein selectively localized at the active zone of presynaptic nerve terminals. J Cell Biol.

[B212] Angenstein F, Niessen HG, Goldschmidt J, Lison H, Altrock WD, Gundelfinger ED, Scheich H (2006). Manganese-Enhanced MRI Reveals Structural and Functional Changes in the Cortex of Bassoon Mutant Mice. Cereb Cortex.

[B213] Eagleson KL, Fairfull LD, Salton SR, Levitt P (2001). Regional differences in neurotrophin availability regulate selective expression of VGF in the developing limbic cortex. J Neurosci.

[B214] Hawley RJ, Scheibe RJ, Wagner JA (1992). NGF induces the expression of the VGF gene through a cAMP response element. J Neurosci.

[B215] Benson DL, Salton SR (1996). Expression and polarization of VGF in developing hippocampal neurons. Brain Res Dev Brain Res.

[B216] Snyder SE, Pintar JE, Salton SR (1998). Developmental expression of VGF mRNA in the prenatal and postnatal rat. J Comp Neurol.

[B217] Salton SR, Ferri GL, Hahm S, Snyder SE, Wilson AJ, Possenti R, Levi A (2000). a novel role for this neuronal and neuroendocrine polypeptide in the regulation of energy balance. Front Neuroendocrinol.

[B218] Ali RR (2004). Prospects for gene therapy. Novartis Found Symp.

[B219] Kicic A, Shen WY, Wilson AS, Constable IJ, Robertson T, Rakoczy PE (2003). Differentiation of marrow stromal cells into photoreceptors in the rat eye. J Neurosci.

[B220] Korhonen L, Brannvall K, Skoglosa Y, Lindholm D (2003). Tumor suppressor gene BRCA-1 is expressed by embryonic and adult neural stem cells and involved in cell proliferation. J Neurosci Res.

[B221] Klingelhutz AJ, Hedrick L, Cho KR, McDougall JK (1995). The DCC gene suppresses the malignant phenotype of transformed human epithelial cells. Oncogene.

[B222] Fearon ER (1996). DCC: is there a connection between tumorigenesis and cell guidance molecules?. Biochim Biophys Acta.

[B223] Keino-Masu K, Masu M, Hinck L, Leonardo ED, Chan SS, Culotti JG, Tessier-Lavigne M (1996). Deleted in Colorectal Cancer (DCC) encodes a netrin receptor. Cell.

[B224] Astic L, Pellier-Monnin V, Saucier D, Charrier C, Mehlen P (2002). Expression of netrin-1 and netrin-1 receptor, DCC, in the rat olfactory nerve pathway during development and axonal regeneration. Neuroscience.

[B225] Forcet C, Stein E, Pays L, Corset V, Llambi F, Tessier-Lavigne M, Mehlen P (2002). Netrin-1-mediated axon outgrowth requires deleted in colorectal cancer-dependent MAPK activation. Nature.

[B226] Tsai HH, Tessier-Lavigne M, Miller RH (2003). Netrin 1 mediates spinal cord oligodendrocyte precursor dispersal. Development.

[B227] Jarjour AA, Manitt C, Moore SW, Thompson KM, Yuh SJ, Kennedy TE (2003). Netrin-1 is a chemorepellent for oligodendrocyte precursor cells in the embryonic spinal cord. J Neurosci.

[B228] Wilson BD, Ii M, Park KW, Suli A, Sorensen LK, Larrieu-Lahargue F, Urness LD, Suh W, Asai J, Kock GA (2006). Netrins promote developmental and therapeutic angiogenesis.. Science.

[B229] Grewal TS, Genever PG, Brabbs AC, Birch M, Skerry TM (2000). Best5: a novel interferon-inducible gene expressed during bone formation. Faseb J.

[B230] Grinspan JB, Edell E, Carpio DF, Beesley JS, Lavy L, Pleasure D, Golden JA (2000). Stage-specific effects of bone morphogenetic proteins on the oligodendrocyte lineage. J Neurobiol.

[B231] Yanagisawa M, Takizawa T, Ochiai W, Uemura A, Nakashima K, Taga T (2001). Fate alteration of neuroepithelial cells from neurogenesis to astrocytogenesis by bone morphogenetic proteins. Neurosci Res.

